# Human mobility acts as a lever for decarbonising wastewater treatment plants

**DOI:** 10.1016/j.ese.2026.100756

**Published:** 2026-08-20

**Authors:** Yiming Meng, Yu Tian, Yu Tao, Shupeng Wang, Purui Wang, Mengxin Li, Lingchao Kong, Andy Deng, Huihang Sun, Wei Zhan, Jiang Yu, Xindan Fan, Yong Tian, Chunmiao Zheng

**Affiliations:** a State Key Laboratory of Urban-rural Water Resource and Environment, School of Environment, Harbin Institute of Technology, Harbin, 150090, China; b School of Civil and Environmental Engineering, Harbin Institute of Technology (Shenzhen), Shenzhen, 518055, China; c School of the Environment and Sustainable Engineering, Eastern Institute of Technology, Ningbo, 315200, China; d State Key Laboratory of Soil Pollution Control and Safety, School of Environmental Science and Engineering, Southern University of Science and Technology, Shenzhen, 518055, China; e School of Mechanical and Manufacturing Engineering, University of New South Wales, Sydney, NSW, 2052, Australia; f Guanlan River Basin Management Center, Water Authority of Shenzhen Municipality, Shenzhen, 518110, China

**Keywords:** Human mobility, Wastewater treatment plants, Greenhouse gas emissions, Decarbonisation, Leverage effect

## Abstract

Wastewater treatment is a substantial and growing source of global greenhouse gas emissions as urban infrastructure expands to meet rising demand and stricter standards. In rapidly urbanising nations such as China, where treatment capacity has more than doubled in the past decade, operational inefficiencies caused by mismatched influent flows and fixed plant capacity represent a major but underexploited decarbonisation opportunity. Conventional load rate metrics systematically overestimate true alignment between wastewater generation and treatment capacity, masking the spatiotemporal mismatches created by intense human mobility. Here we demonstrate that human mobility acts as a powerful yet previously overlooked lever for decarbonising wastewater treatment plants. Using more than 170 million high-resolution population heatmap records from Shenzhen, a megacity in southern China with a highly mobile population exceeding 17 million, we show that Spring Festival outflows reduce citywide population presence by 64%, lowering the treatment and inflow dynamic equilibrium (TIDE) index by 23% and raising greenhouse gas emission intensity by 28%, whereas intercity commuting improves alignment and cuts emission intensity by 27%. Two mobility-informed operational strategies—gradient shutdown during holidays and flow equalisation under routine conditions—reduce plant-level emissions by up to 18% and offer an average 22% improvement in TIDE across Chinese cities. These findings establish human mobility as a scalable, low-capital lever for aligning wastewater infrastructure with real behavioural dynamics and advancing climate mitigation goals.

## Highlights

• Human mobility acts as a powerful lever for decarbonising wastewater treatment plants.
• Spring Festival outflows raise emission intensity by 28% through worsened alignment.
• Intercity commuting improves alignment and cuts emission intensity by 27%.
• Mobility-informed strategies cut plant-level emissions by up to 18% with 22% national gains.

## Graphical abstract

**Figure:**
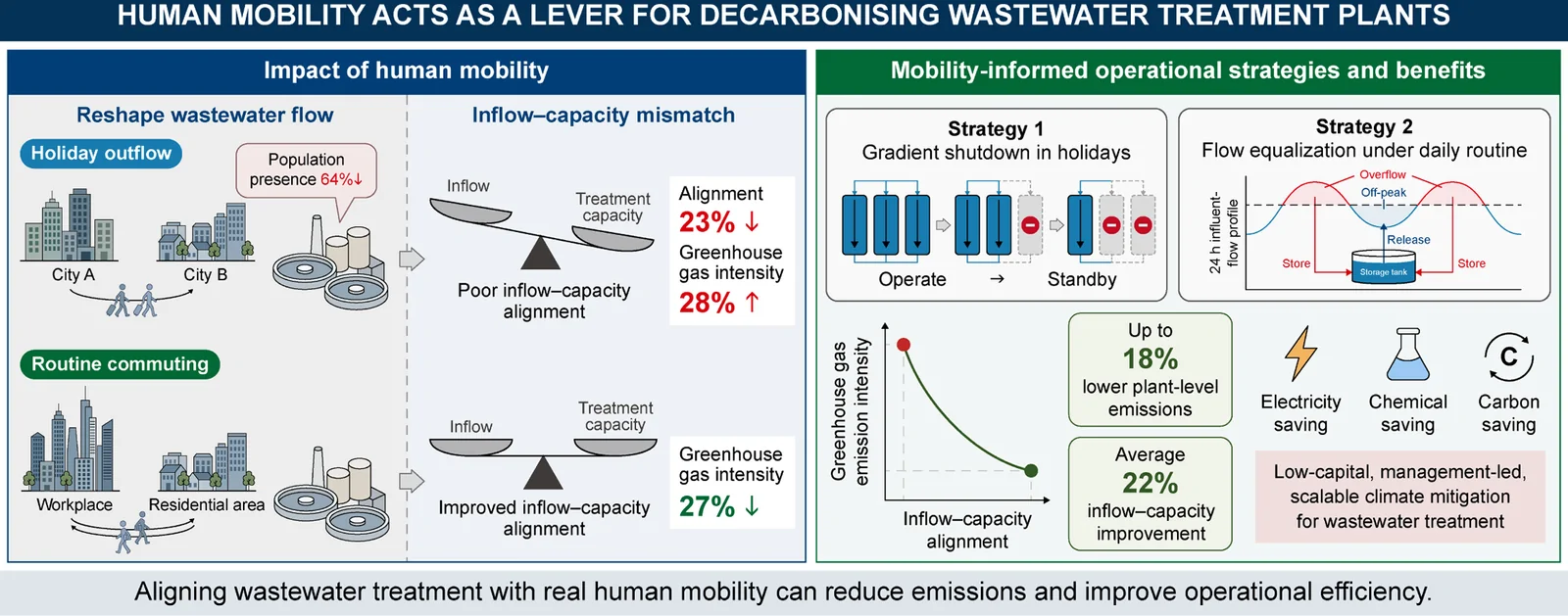

## 1 Introduction

The wastewater sector is energy-intensive and a major source of greenhouse gas (GHG) emissions, and therefore has substantial potential to contribute to climate change mitigation [[1], [2], [3], [4]]. Indeed, the sector is estimated to account for nearly 2% of global GHG emissions [5]. Yet, growing treatment demand and progressively stricter effluent standards are expected to further increase sectoral emissions worldwide [6]. China has the world's largest wastewater treatment infrastructure, which continues to expand. From 2009 to 2019, the number of urban wastewater treatment plants (WWTPs) in China increased from 1214 to 2471 [7], while the sector's GHG emissions rose from 22.4 to 53.0 Mt carbon dioxide equivalent (CO_2_-eq), 2.4 times the 2009 level [3]. Decarbonising the wastewater sector is therefore pivotal to achieving China's dual carbon goals—peaking carbon dioxide emissions before 2030 and achieving carbon neutrality before 2060.

Emerging evidence indicates that optimising operational control to better align influent flow with treatment capacity can deliver substantial energy and cost savings while also reducing GHG emissions [8,9]. This opportunity reflects an under-recognised operational lever: WWTPs use less electricity and fewer chemicals per unit of treated water when the influent flow approaches design capacity [10,11]. As a result, improving the treatment–inflow alignment can reduce emissions from treatment processes. Based on data from China's National Pollution Source Census, Niu et al. [12] report that fully loaded WWTPs consume approximately 12% less electricity than underloaded or overloaded plants, while increasing the WWTP loading could save more energy (5.9%) than renovating old WWTPs (3.2%). Operational optimisation that improves treatment–inflow alignment could therefore reduce GHG emissions without requiring capital-intensive retrofits. Studies have explored the decarbonisation potential of real-time intelligent control in WWTPs by using time-varying influent and process information to guide operational optimisation. Such studies mainly focus on dynamically storing or shifting influent flows to support flow equalisation [8,13] or on using predictions of influent and process parameters to optimise aeration or chemical dosing [14,15]. However, these studies rarely consider how dynamic population mobility affects fluctuations in domestic wastewater or how these effects propagate to treatment-related GHG emissions. This gap in the literature largely stems from the lack of real-time population mobility information, which results in the decarbonisation potential of improving treatment–inflow alignment being insufficiently evaluated.

Considering the relatively fixed capacity of WWTPs, population-driven fluctuations in domestic wastewater are an important driver of treatment–inflow alignment, making dynamic population presence a useful operational signal for alignment optimisation. But driven by activities such as work, study, and recreation, the actual population presence exhibits both periodic and aperiodic fluctuations, introducing substantial uncertainty. First, the spatial separation between workplaces and residences leads residents to engage in daily round-trip commuting or in weekly weekday departures and weekend returns [16]. For instance, in China's Greater Bay Area, intercity travel averaged 7.2 million trips per day in 2024 and continues to increase [17]. Second, irregular movements associated with holidays, medical visits, or business travel further perturb local population baselines. For example, during the 40-day Spring Festival travel rush in 2023, associated trips totalled 4.7 billion nationwide [18]. Similar high-intensity mobility occurs in many global metropolises, including New York, Tokyo, Seoul, and London, as well as during major festive periods such as Christmas, Songkran, and Diwali [19,20]. This mobility reshapes the spatiotemporal distribution of wastewater, altering the treatment–inflow alignment and markedly influencing GHG emissions from WWTPs. Additionally, wastewater treatment planning has long relied on static, residence-based census data because of their official status, standardisation, and wide availability. However, such data mask the spatiotemporal mismatches exacerbated by intense human mobility [[21], [22], [23]], rendering them ineffective as an operational signal for guiding alignment optimisation.

Emerging large-scale spatiotemporal datasets offer new opportunities to track dynamic population changes [24]. These include social media activity signals, such as Twitter and Weibo, and location-based navigation data, such as Google Maps and Baidu Maps. Such datasets can characterise human mobility at a microscopic scale, providing unprecedented spatiotemporal resolution for understanding population dynamics [[25], [26], [27], [28], [29]]. They have also been applied in environmental and public health studies as proxies for mobility when assessing exposure to air pollution [30,31], coronavirus disease 2019 (COVID-19) [[32], [33], [34], [35]], and natural disasters such as floods and hurricanes [27,36]. Nevertheless, to our knowledge, such data have not been directly linked to WWTP operational performance. It therefore remains unclear to what extent distinct mobility patterns alter treatment–inflow alignment and how this effect translates into changes in GHG emissions from WWTPs.

This study quantifies the decarbonisation potential of mobility-informed optimisation of treatment–inflow alignment using large-scale population mobility data. First, we develop the treatment and inflow dynamic equilibrium (TIDE) index to flexibly evaluate the treatment–inflow alignment across multiple spatiotemporal scales (hourly, daily, and annual temporal scales; plant, regional, and city spatial scales), thereby linking human mobility to GHG emissions from WWTPs. Building on this, using over 170 million population heatmap records and 320,000 hourly influent observations from Shenzhen, China, we characterise the differential response mechanisms of population mobility, treatment–inflow alignment, and GHG emissions across three distinct patterns: Spring Festival homecoming, daily intercity mobility, and daily interdistrict commuting. Finally, we propose two mobility-informed decarbonisation strategies tailored for both routine and holiday scenarios, validate their mitigation effects using the Longhua Phase 1 WWTP in Shenzhen as a representative case, and assess their scalability across more than 350 cities nationwide. This study extends population mobility analysis to the operational decarbonisation of WWTPs. Its findings provide insights into the human–water–climate nexus and indicate an alternative operational pathway for WWTP decarbonisation alongside conventional technological upgrades.

## 2 Materials and methods

### 2.1 Mobility data

The spatiotemporal population distribution and mobility were characterised using population heatmap data obtained from Baidu Huiyan (https://huiyan.baidu.com/), a spatiotemporal big data service built on Baidu Maps that provides aggregated analytics based on large-scale anonymised location-based service (LBS) requests generated by mobile devices. The estimates are derived from over 120 billion LBS enquiries per day made from approximately 1.1 billion mobile devices [37]. Baidu Maps supports location services for more than 650,000 applications and websites each day, accounting for around 75% of the domestic map service market. Although these data are not a direct measure of all human activity, they can capture broader patterns in population spatial distribution and mobility trends.

We obtained more than 170 million hourly population heatmap records from Baidu Huiyan from January 1 to December 31, 2023. This period was selected because citywide hourly WWTP influent data and Baidu Huiyan heatmap data were both available for the same interval. Each record consisted of a geographic coordinate and an associated population heat value, representing the relative population concentration within a 200 × 200 m grid cell centred on the given coordinate. We also collected daily population migration data from Baidu Huiyan for 369 cities across China from 2021 to 2023. We used the inflow and outflow indices to represent the relative intensity of the daily incoming and outgoing populations, respectively, for each city or region at the national scale.

### 2.2 Quantifying the TIDE across different scales

China has expanded its wastewater treatment facilities substantially over the past two decades [38]. However, it remains unclear whether these facilities have been fully utilised and whether their capacity matches regional domestic wastewater generation.

Operational efficiency is commonly assessed via the load rate (L):

(1) $$L=\frac{\sum_{r}Q_{r}}{\sum_{r}D_{r}}$$

where $Q_{r}$ represents the daily influent volume received and treated by region r (10^4^ m^3^ day^−1^), $D_{r}$ represents the corresponding total daily design treatment capacity (10^4^ m^3^ day^−1^), $\sum_{r}Q_{r}$ is the total daily influent flow, and $\sum_{r}D_{r}$ is the total designed treatment capacity.

**Fig. 1:**
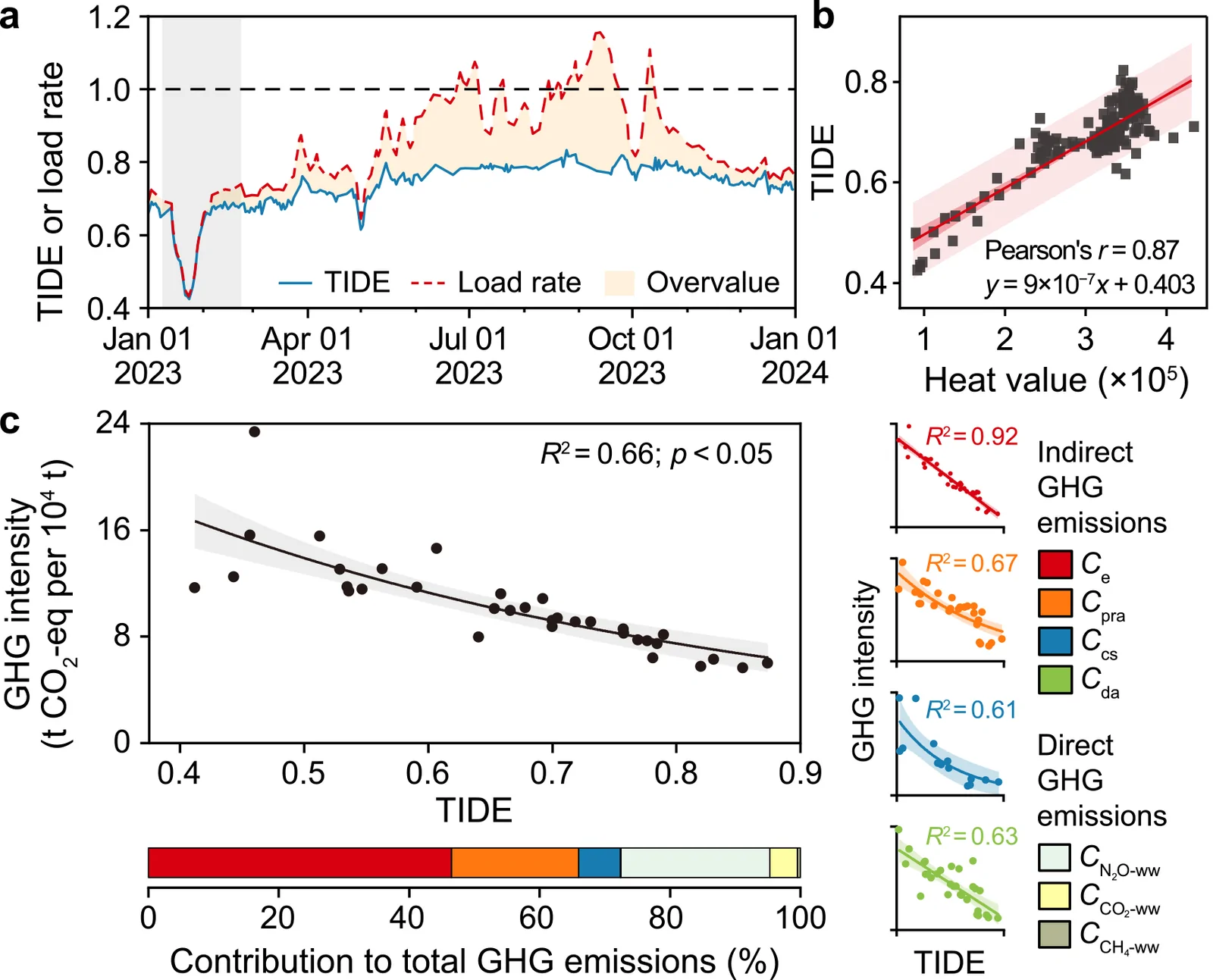
**Dynamic treatment–inflow alignment and its relationship with population and greenhouse-gas emissions in Shenzhen. a**, Daily treatment and inflow dynamic equilibrium (TIDE) index and conventional load rate during 2023. The yellow shading highlights the overestimation of alignment by the load rate metric, and the grey shading denotes the Spring Festival travel rush from January 7 to February 15, 2023. The horizontal dashed line indicates perfect treatment–inflow alignment (TIDE = 1); for the load rate, a value of 1 indicates that total influent flow equals total design capacity. **b**, Relationship between daily population heat values and TIDE in Shenzhen. The red line represents the fitted linear regression, while the dark and light shaded bands indicate the 95% confidence interval and 95% prediction interval, respectively. **c**, Relationship between TIDE and greenhouse gas (GHG) intensity (t CO_2_-eq per 10^4^ t wastewater) at the representative wastewater treatment plant. The four plots on the right show the relationships between TIDE and the GHG intensities associated with indirect emission sources, including electricity use ($C_{e}$), phosphorus-removal agents ($C_{\mathrm{pra}}$), external carbon source ($C_{\mathrm{cs}}$), and dewatering agents ($C_{\mathrm{da}}$), from top to bottom. Each point represents one monthly observation; the solid lines show the fitted regression, and the shaded bands indicate the 95% confidence intervals. The stacked bar shows the percentage contributions of indirect and direct emission sources to total treatment-related GHG emissions. $C_{e}$, $C_{\mathrm{pra}}$, $C_{\mathrm{cs}}$, and $C_{\mathrm{da}}$ contributed 46.5%, 19.5%, 6.4%, and 0.1%, respectively. Direct emission sources, including N_2_O emissions from total nitrogen removal ($C_{N_{2}O-\mathrm{ww}}$), CO_2_ emissions from the complete oxidation of external carbon sources ($C_{CO_{2}-\mathrm{ww}}$), and CH_4_ emissions from organic matter removal ($C_{CH_{4}-\mathrm{ww}}$), contributed 22.8%, 4.3%, and 0.5%, respectively.

Yet this metric substantially underestimates the mismatches between wastewater generation and treatment capacity, primarily because it neglects the spatial and temporal heterogeneity of wastewater distribution and differences in design capacity among WWTPs (Fig. 1a).

To address this issue, we proposed the TIDE index (T), using hourly influent data from WWTPs to flexibly quantify treatment–inflow alignment across multiple spatial and temporal scales. TIDE can be calculated at the hourly, daily, or annual temporal scale at the plant, regional, or city spatial scale. At the city or regional level, daily TIDE was calculated by aggregating plant-level treatment–inflow alignment using weights proportional to the design capacity of each plant:

(2) $$T=1-\frac{\sum_{i}\sum_{t}\left|\frac{q_{i,t}}{d_{i}}-1\right|\times \omega_{i}}{24}$$

where $q_{i,t}$ is the hourly influent volume received and treated by plant i at hour t (10^4^ m^3^ h^−1^), $d_{i}$ is the plant's hourly design capacity ($d_{i}=D_{i}/24\text{;}$ 10^4^ m^3^ h^−1^), and $\omega_{i}$ is its design-capacity weight. This weighting accounts for differences in treatment scale among plants and is defined as:

(3) $$\omega_{i}=\frac{D_{i}}{\sum_{i}D_{i}}$$

Regional TIDE at hour t was calculated as the design-capacity-weighted aggregation of plant-level treatment–inflow alignment:

(4) $$T_{t}=1-\sum_{i}\left|\frac{q_{i,t}}{d_{i}}-1\right|\times \omega_{i}$$

At the individual-plant level, daily TIDE was calculated as the mean of the 24 hourly values (equation (5)), where the corresponding hourly value for plant i was calculated in equation (6):

(5) $$T_{i}=1-\frac{\sum_{t}\left|\frac{q_{i,t}}{d_{i}}-1\right|}{24}$$

(6) $$T_{i,t}=1-\left|\frac{q_{i,t}}{d_{i}}-1\right|$$

To minimise the influence of rainfall-driven infiltration and inflow, the daily analyses for Shenzhen were limited to 243 rain-free days in 2023. To evaluate the decarbonisation potential at the national level, we assessed the TIDE for 369 cities across China from 2021 to 2023. As hourly influent data were not available for all WWTPs, we adopted a top-down allocation approach. Annual city-level treatment volumes from the *China Urban Construction Statistical Yearbook* were first distributed across days using the daily population heatmap values obtained from Baidu Huiyan (Supplementary Fig. S1). Hourly distributions were then determined using a wastewater-generation coefficient inventory. The regional classification followed the *Technical Manual for Pollution Discharge Coefficients of Domestic Sources* from the Second National Pollution Census, which divides China into six zones according to geographical, economic, climatic, and water-use characteristics (Supplementary Fig. S2). We assumed uniform hourly discharge profiles within each region, based on empirical monitoring at residential sites (Supplementary Table S1). Each city was treated as a virtual treatment plant with a total annual volume and combined design capacity. Hourly and daily city-level TIDE values were then calculated using equations (6) and (5), respectively.

### 2.3 Accounting for the GHG emissions of WWTPs

In this study, GHG emissions from WWTPs were defined to comprise treatment-process-related emissions and exclude emissions from final sludge disposal. The GHG accounting framework focused on direct and indirect GHG emissions associated with the wastewater treatment process. This boundary was adopted because the final sludge disposal emissions are primarily determined by sludge yield and the subsequent disposal pathway, whereas the proposed operational strategies primarily redistributed the hourly influent flow and improved treatment load matching without directly changing the final sludge yield or the sludge disposal pathway. The treatment process emissions comprised three categories of direct emissions: methane emissions from organic matter removal, nitrous oxide emissions from total nitrogen removal, and carbon dioxide emissions from the complete oxidation of external carbon sources. They also included four categories of indirect emissions: emissions from electricity use, phosphorus removal agents, dewatering agents, and external carbon sources.

We used an emission factor method to estimate GHG emissions. CH_4_ emissions were converted to CO_2_-eq using a 100-year global warming potential (GWP) of 28 t CO_2_-eq per t CH_4_, whereas N_2_O emissions were converted using a 100-year GWP of 265 t CO_2_-eq per t N_2_O, as reported in the Intergovernmental Panel on Climate Change's *Climate Change 2014: Synthesis Report* [39].

Direct CH_4_ emissions from wastewater treatment ($C_{{\text{CH}}_{4}-\text{ww}}$; t CO_2_-eq) primarily occur during anaerobic processes in units such as primary sedimentation tanks and biological treatment stages, and were calculated as follows:

(7) $$C_{CH_{4}-\text{ww}}=\left[\left(R_{\text{COD}}-S\times \rho_{s}\right)\times B_{o}{\times M}_{\text{CF}}-\omega_{CH_{4}}\right]\times V{\times G}_{CH_{4}}$$

where $R_{\text{COD}}$ represents the amount of chemical oxygen demand (COD) removed per unit volume of treated wastewater (t COD m^−3^); S represents dry sludge production per unit volume of treated wastewater (t m^−3^); $\rho_{s}$ represents the amount of organic matter contained in the sludge (t COD t^−1^); $B_{o}$ represents the producing capacity of methane (t CH_4_ per t COD); $M_{\text{CF}}$ represents the methane correction factor; $\omega_{CH_{4}}$ represents the amount of methane recovered per unit volume of treated wastewater (t CH_4_ m^−3^); V represents the total volume of wastewater treated during the accounting period (m^3^); $G_{CH_{4}}$ represents the global warming potential of methane (t CO_2_-eq per t CH_4_). The default values of $B_{o}$, $M_{\text{CF}}$, and $G_{CH_{4}}$ were adopted from the *Technical specification for low-carbon operation evaluation of sewage treatment plant* [40], with values of 0.25 t CH_4_ per t COD, 0.003, and 28 t CO_2_-eq per t CH_4_, respectively. Because most WWTPs in Shenzhen lacked methane recovery systems, $\omega_{CH_{4}}$ was assumed to be zero.

Direct N_2_O emissions from biological treatment units ($C_{N_{2}O-\text{ww}}$; t CO_2_-eq) were calculated as follows:

(8) $$C_{N_{2}O-\text{ww}}=R_{\text{TN}}{\times E}_{N_{2}O}{\times C}_{N_{2}O/N_{2}}\times V{\times G}_{N_{2}O}$$

where $R_{\text{TN}}$ represents the amount of total nitrogen removed per unit volume of treated wastewater (t TN m^−3^); $E_{N_{2}O}$ represents the emission factor for nitrous oxide (0.016 t N_2_O-N per t TN); $C_{N_{2}O/N_{2}}$ represents the conversion factor from N_2_O-N to N_2_O (44/28 t N_2_O per t N_2_O-N); and $G_{N_{2}O}$ represents the global warming potential of nitrous oxide (265 t CO_2_-eq per t N_2_O).

External carbon added for denitrification was assumed to be completely oxidised to CO_2_. The associated direct CO_2_ emissions ($C_{{\text{CO}}_{2}-\text{ww}}$; t CO_2_-eq) were estimated as follows:

(9) $$C_{{\text{CO}}_{2}-\text{ww}}=\sum_{j}\left(y_{\text{cs},j}{\times E}_{CO_{2},j}{\times G}_{CO_{2}}\right)$$

where $y_{\text{cs},j}$ represents the consumption of the external carbon source j (t) and $E_{CO_{2},j}$ represents the CO_2_ emission factor for the conversion of external carbon source j (t CO_2_ t^−1^; 1.07 t CO_2_ t^−1^ for CH_3_COONa, 1.47 t CO_2_ t^−1^ for CH_3_OH and other external carbon sources, following Zhou et al. [41]). $G_{CO_{2}}$ represents the global warming potential of CO_2_ (1 t CO_2_-eq per t CO_2_).

Indirect GHG emissions are associated with the consumption of electricity, heat, and chemicals. Because electricity was the only operational energy source reported for most WWTPs in the Shenzhen dataset, the energy-related analysis was restricted to electricity consumption. The corresponding indirect GHG emissions ($C_{e}$; t CO_2_-eq) were calculated as follows:

(10) $$C_{e}=E_{H}\times E_{{\text{CO}}_{2},e}\times G_{{\text{CO}}_{2}}$$

where $E_{H}$ represents the electricity consumption of the individual WWTP (kWh) and $E_{{\text{CO}}_{2},e}$ represents the CO_2_ emission factor for electricity consumption (8.042 × 10^−4^ t CO_2_ kWh^−1^ for the Shenzhen municipal grid) [40].

Indirect GHG emissions associated with chemical inputs ($C_{x}$; t CO_2_-eq), including phosphorus-removal agents, dewatering agents, and external carbon sources, were calculated using a unified formulation:

(11) $$C_{x}=\sum_{k=1}^{n_{x}}\left(y_{x,k}\times E_{CO_{2},x,k}\times G_{CO_{2}}\right)$$

where *x* represents phosphorus-removal agents, dewatering agents, or external carbon sources; $n_{x}$ represents the number of chemical inputs in category x; $y_{x,k}$ represents the consumption of input k (t); $E_{CO_{2},x,k}$ represents the corresponding CO_2_ emission factor (t CO_2_ t^−1^). The calculations used chemical-specific CO_2_ emission factors drawn from the relevant references (Supplementary Table S2).

The GHG emission intensity ($I_{\text{GHG}}$; t CO_2_-eq per 10^4^ t wastewater) was defined as the total GHG emissions per unit of treated wastewater and calculated as follows:

(12) $$I_{\text{GHG}}=\frac{C_{CH_{4}-\text{ww}}+C_{N_{2}O-\text{ww}}+C_{CO_{2}-\text{ww}}+C_{e}+\sum_{x}C_{x}}{W}$$

where W represents the amount of wastewater treated during the accounting period (10^4^ t).

## 3 Results and discussion

### 3.1 A leverage effect between treatment–inflow alignment and GHG emissions

We developed the TIDE index to flexibly assess whether wastewater generation is dynamically aligned with the available treatment capacity (Section 2.2). TIDE ranges from −∞ to 1, with higher values indicating better alignment between influent flow and treatment capacity. We first applied TIDE to Shenzhen, a megacity in southern China (Supplementary Text S1), and subsequently extended the analysis nationwide.

The conventional load rate overestimated treatment–inflow alignment in Shenzhen. This metric has long been used as a rough proxy for treatment–inflow alignment (Section 2.2). However, our results showed that the citywide load rate in Shenzhen in 2023 exceeded the TIDE index by an average of 10.8%, with a maximum overestimation of 50.6% (Fig. 1a). This overestimation can be illustrated using a conceptual example (Supplementary Fig. S3). In this example, three hypothetical regions have identical load rates of 1 but TIDE values of 0, 1, and 0, respectively, reflecting differences in the temporal distribution of influent and their allocation of loads among plants. While both the traditional load rate and the TIDE index reflect the treatment–inflow alignment, the TIDE index explicitly captures the spatiotemporal accumulation of mismatches and incorporates the plant-scale heterogeneity. It therefore offers a more accurate representation of the treatment–inflow alignment across the wastewater system.

Consistent with previous research [10,11], higher TIDE values are associated with lower GHG emission intensity in wastewater treatment (Fig. 1c; see Section 2.3 for the calculation of GHG emissions and emission intensity). Population mobility reshapes the actual population presence within WWTP service areas, thereby changing the hourly influent flow and TIDE. As influent flow approaches the design capacity (i.e., as TIDE approaches 1), WWTPs operate closer to their design loading conditions, reducing electricity and chemical consumption per unit of treated wastewater and lowering indirect GHG emission intensity. Using nearly three years of monthly operational data from 35 WWTPs in Shenzhen (January 2022 to October 2024), we further decomposed total GHG emissions and used plant-specific analyses to explain why higher TIDE is associated with lower GHG emission intensity. In this regard, indirect emissions dominated the treatment process, accounting for over 72% of the total GHG emissions (Fig. 1c, lower panel). Moreover, across the individual WWTPs, all four categories of indirect emissions generally exhibited negative relations with the TIDE (Fig. 1c, right panel; Supplementary Table S3). These findings reveal a previously overlooked decarbonisation pathway—namely, that under a constant treatment volume, improving the TIDE can effectively reduce GHG emissions from the treatment process by improving energy and chemical use efficiency.

Given that wastewater treatment capacity is generally fixed, population dynamics can drive fluctuations in domestic wastewater generation, thereby affecting TIDE. We characterised these dynamics using Baidu Huiyan population heatmap data as a proxy for the relative population presence (Section 2.1). At the city scale, TIDE is positively correlated with the population heat value (*r* = 0.87, *p* < 0.01), with a stronger association at lower population levels (Fig. 1b). This finding suggests that, at the city scale, larger in-city populations improve the alignment between wastewater generation and available treatment capacity. As TIDE approaches 1, the GHG emission intensity of wastewater treatment is expected to decline.

### 3.2 Spring Festival outflow reduces the TIDE and boosts the GHG emission intensity

China's Spring Festival travel rush, also known as chunyun, is often described as the world's largest annual human migration [37]. In 2023, over 4.7 billion passenger trips were recorded within just 40 days via railways, highways, waterways, and civil aviation [18]. Shenzhen has a particularly large migrant population: according to the Statistics Bureau of Shenzhen Municipality, in 2023, the city was home to 11.7 million residents without local household registration, representing 65.9% of its total population [42]. Against this demographic background, the Spring Festival travel rush was accompanied by a substantial outflow and coincided with the lowest citywide TIDE observed during the study period (Fig. 1a, grey shading).

**Fig. 2:**
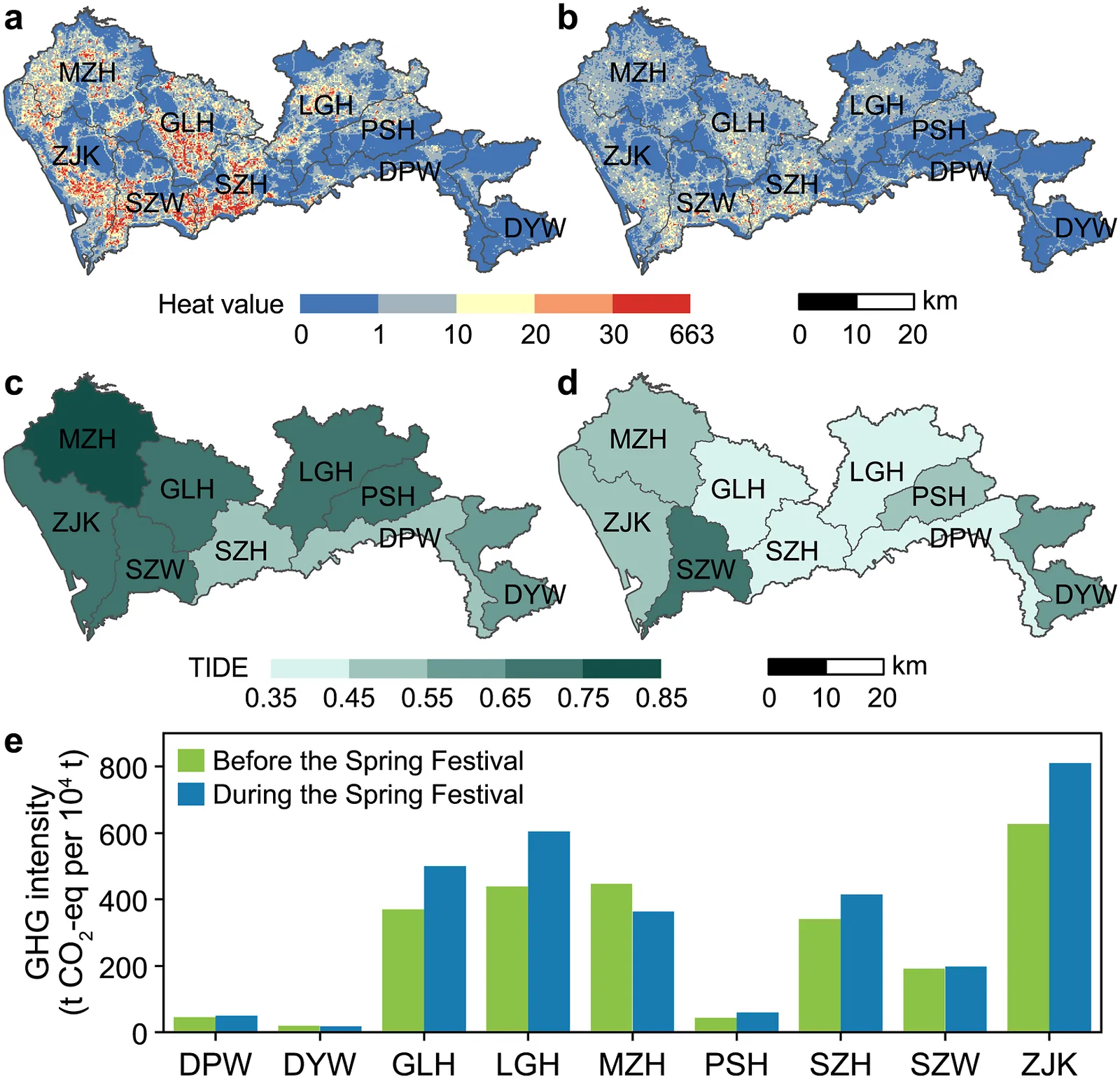
**Effects of Spring Festival population outflow on treatment–inflow alignment and greenhouse-gas emissions in Shenzhen. a**–**b**, Population heat-value distributions at a spatial resolution of 200 × 200 m before and during the Spring Festival on January 5, 2023 (a typical working day before the Spring Festival; **a**), and January 22, 2023 (the day of Chinese New Year; **b**). MZH, GLH, SZH, LGH, and PSH denote the Maozhou, Guanlan, Shenzhen, Longgang, and Pingshan River basins, respectively; ZJK, SZW, DPW, and DYW denote the Pearl River Estuary, Shenzhen Bay, Dapeng Bay, and Daya Bay water systems, respectively. **c**–**d**, Daily treatment and inflow dynamic equilibrium (TIDE) index before the Spring Festival on January 6, 2023 (**c**) and during the Spring Festival on January 23, 2023 (**d**). **e**, Treatment-related greenhouse-gas (GHG) emission intensities in the nine basins before and during the Spring Festival (expressed as t CO_2_-eq per 10^4^ t of wastewater).

Using population heatmap data, we compared Shenzhen's population before (January 5, 2023, a typical working day before the Spring Festival; Fig. 2a) and during (January 22, 2023, the day of Chinese New Year; Fig. 2b) the Spring Festival travel rush. The aggregated population heat value declined from 263,901.8 to 95,527.8, a decrease of 63.8%. In Shenzhen, basins serve as the smallest administrative units for centralised WWTP management, and the city can be divided into nine regions [43]. Across these regions, TIDE decreased by an average of 22.5% during the Spring Festival travel rush (Fig. 2c and d). Over the same period, GHG emission intensity increased by an average of 27.7%, with the largest increase of over 100% observed in the Maozhouhe (MZH) basin (Fig. 2e). These results indicate that the Spring Festival population outflow reduced the treatment–inflow alignment, leading to inefficient WWTP operation and elevated GHG emission intensity.

### 3.3 Intercity commuting delivers GHG mitigation dividends

**Fig. 3:**
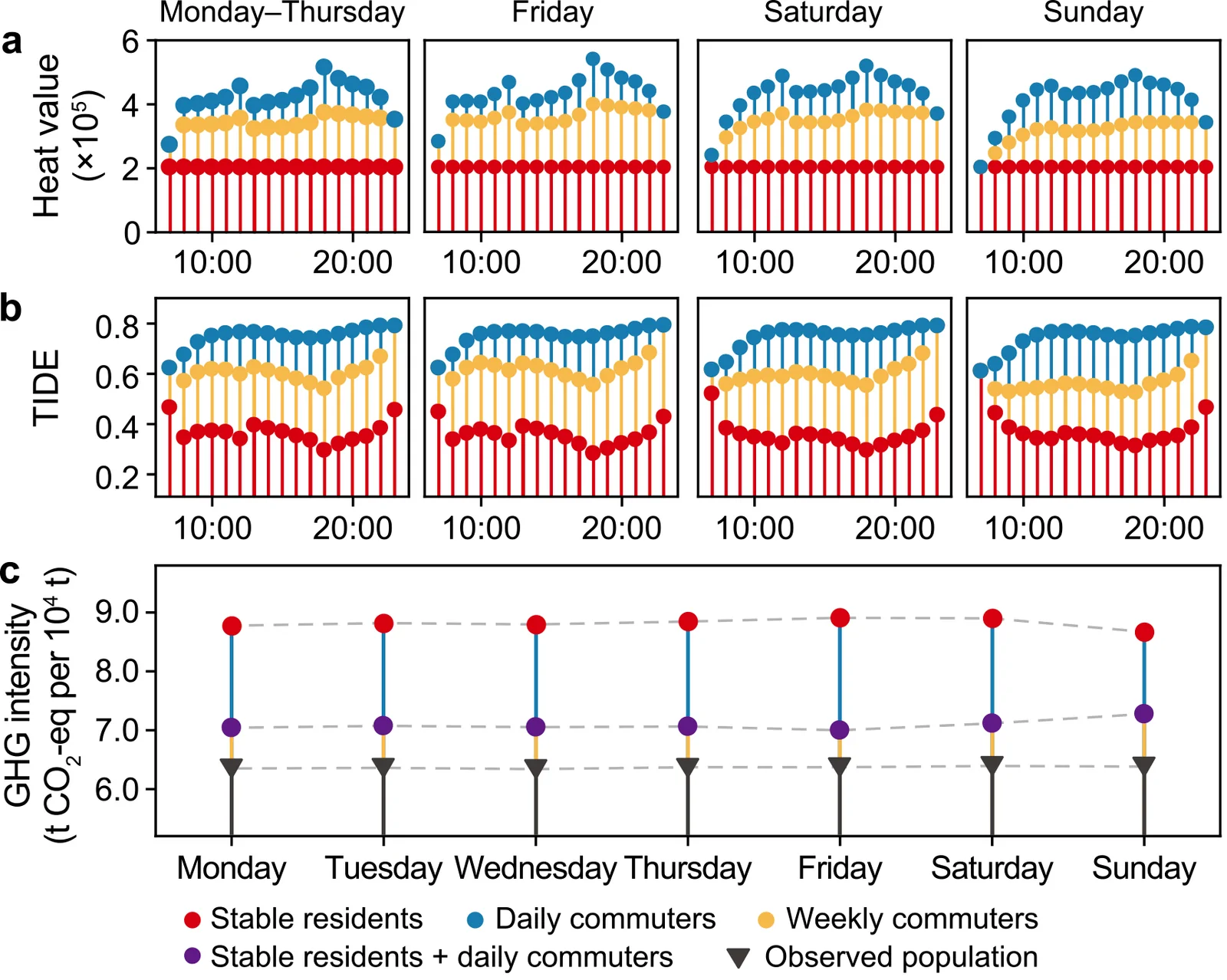
**Effects of commuting-related population dynamics on treatment–inflow alignment and greenhouse-gas emissions. a**–**b**, Hourly population heat values (**a**) and corresponding treatment and inflow dynamic equilibrium (TIDE) index (**b**) from 07:00 to 23:00 on Monday–Thursday, Friday, Saturday, and Sunday, based on 5832 hourly observations. Blue, yellow, and red lollipops represent daily commuters, weekly commuters, and stable residents, respectively, with stem length indicating each group's contribution to the total value. **c**, Treatment-related greenhouse-gas (GHG) emission intensity across the week under three population compositions. Red circles, purple circles, and black inverted triangles represent stable residents only, stable residents and daily commuters, and the observed population (stable residents, daily commuters, and weekly commuters), respectively. The blue solid segments indicate the reduction in GHG emission intensity attributable to daily commuters, whereas the yellow solid segments indicate the reduction attributable to weekly commuters. Dashed lines connect estimates for the same population composition across days.

Given frequent intercity population flows within major urban agglomerations, we also examined the impact of intercity commuting on Shenzhen's wastewater system (Fig. 3). Our method decomposed the urban population into three categories: daily commuters, weekly commuters, and stable residents (Supplementary Text S2; Fig. 3a). This classification further allowed us to quantify the contributions of each group to the citywide population and TIDE (Fig. 3a and b).

Scenario analysis separated the GHG effects of daily and weekly intercity commuting (Fig. 3c). Overall, intercity commuting delivers GHG mitigation dividends to Shenzhen, reducing the average GHG emission intensity by 27.2%. This reduction is consistent with the fact that many urban WWTPs in China are designed with surplus capacity to accommodate future population growth (Supplementary Fig. S1). Intercity commuters bring domestic wastewater volumes closer to design capacity, increasing the overall TIDE and thereby reducing GHG emissions (Fig. 3b and c). However, during periods of heavy inflow, WWTPs may exceed capacity during peak sewage generation, leading to a decline in the TIDE and an elevated risk of overflow pollution. Thus, the GHG mitigation benefit of commuting depends on the level of surplus capacity in wastewater treatment systems.

### 3.4 Cross-district commuting reshapes the spatiotemporal distribution of the population and GHG emissions

**Fig. 4:**
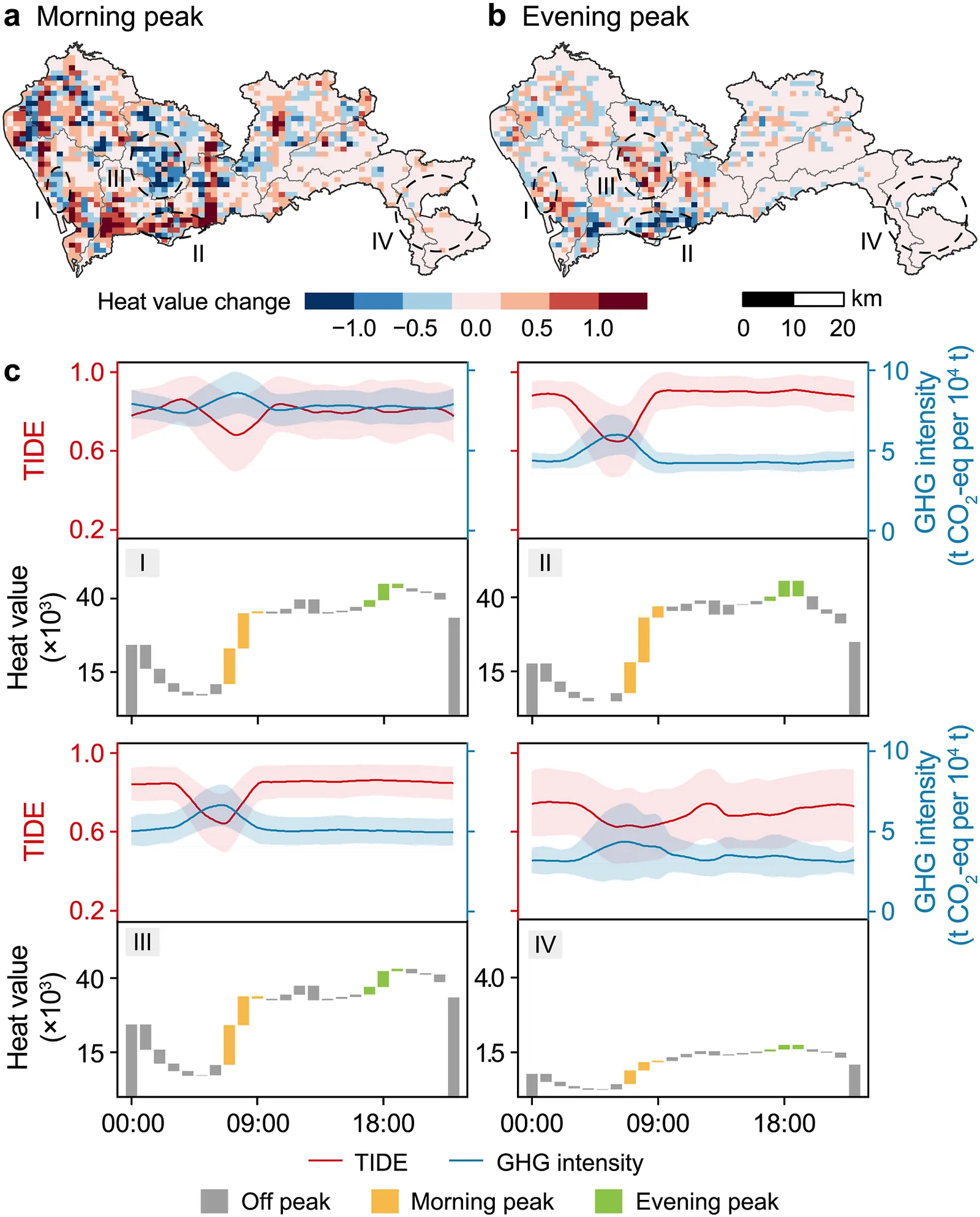
**Spatiotemporal effects of cross-district commuting on population distribution and greenhouse-gas emissions. a**–**b**, Grid-scale population changes during the morning commuting peaks from 07:00 to 10:00 (**a**) and the evening commuting peak from 17:00 to 20:00 (**b**) on November 8, 2023, a representative rain-free weekday not close to any holiday. Population data were aggregated from 200 × 200 m grids to 1 × 1 km grids to enhance visualisation of agglomeration effects, and then *z*-score-transformed to eliminate the impact of inherent daily activity intensity on the population heat value. Four typical spatial clusters (I–IV) were identified based on commuting-driven population shifts. **c**, Hourly changes in population, treatment and inflow dynamic equilibrium (TIDE) index, and treatment-related greenhouse-gas (GHG) intensity across the four identified clusters. Bar colours distinguish off-peak, morning-peak, and evening-peak periods. Lines show TIDE and GHG emission intensity. The shaded bands indicate the mean ± standard deviation.

Spatial separation between residences and workplaces is common in cities [44,45], generating frequent cross-district commuting. Consequently, domestic wastewater generation is not confined to residential areas, but is redistributed through daily mobility. Grid-scale population changes derived from the heatmap data exhibited opposing spatial patterns during the morning (07:00–10:00) and evening (17:00–20:00) commuting peaks, reflecting the daily redistribution of population across districts (Fig. 4a and b). Based on these population heat patterns, we identified four representative zones (I–IV). The four representative zones exhibited distinct weekday diurnal variations in population, TIDE, and GHG emission intensity (Fig. 4c).

The four zones showed different population patterns, consistent with their distinct urban functions. Zone I, characterised by high-density urban villages (e.g., Sanwei and Huangmabu, which are among Shenzhen's top five largest urban villages), experienced a population decline after the morning peak and a rebound after the evening peak. Zone II—home to Shenzhen's four major employment hubs (Chegongmiao, the Futian central business district, Huaqiangbei, and the Luohu Golden Triangle)—absorbed commuters from across the city, resulting in a population increase after the morning peak. Zone III, covering Longhua District, is a multifunctional area near Shenzhen's geographic centre. Most parts of this zone lost population after the morning peak, although increases occurred near smart-manufacturing hubs such as Foxconn and Huawei. Zone IV, a major tourist area, showed limited population changes around the morning and evening peaks.

While population and TIDE covaried at the daily scale (Fig. 1b and 2a–d), their hourly patterns were not fully synchronised. Across the four zones, TIDE consistently reached its minimum between 06:00 and 08:00, approximately 2 h after the population heat value minimum at 04:00–05:00. This lag probably reflects the time required for wastewater generated across the catchment to reach WWTPs. At the hourly scale, GHG emission intensity varied inversely with TIDE across all four zones, consistent with the negative TIDE–GHG emission intensity relationship identified earlier (Fig. 1c).

### 3.5 Mobility-informed decarbonisation strategies

Building on the observed link between population mobility and GHG emissions from WWTPs, we propose two influent-flow optimisation strategies for the Spring Festival and regular daily operating conditions. Both strategies aim to improve treatment–inflow alignment and ultimately reduce GHG emissions from WWTPs.

**Fig. 5:**
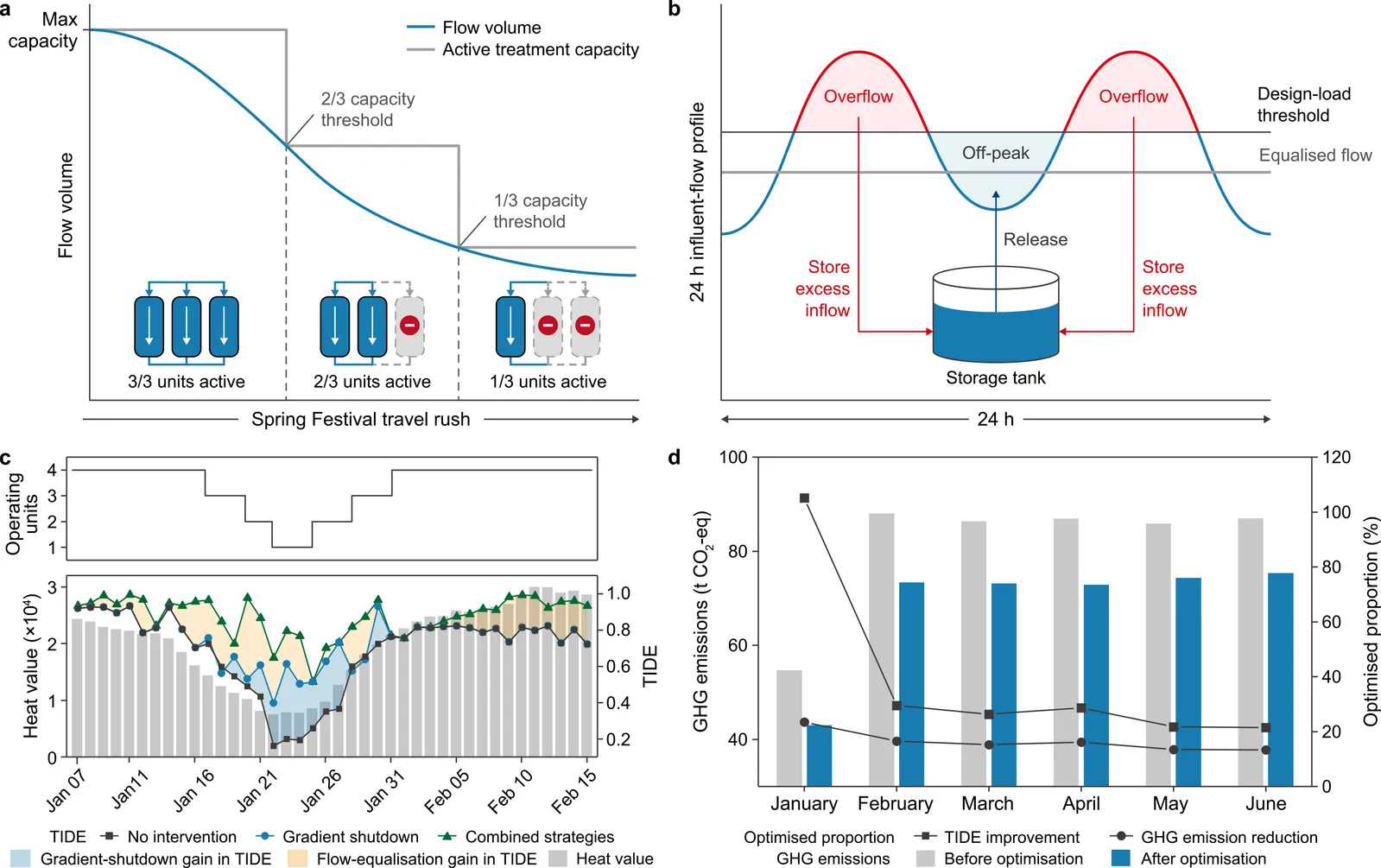
O**perational strategies for improving treatment–inflow alignment and reducing greenhouse-gas emissions. a,** Conceptual gradient-shutdown strategy during the population-outflow phase of the Spring Festival travel rush. As influent flow declines (blue curve), active treatment capacity (grey stepped line) is reduced from three to two, then to one parallel treatment unit, when flow reaches two-thirds and one-third of the design capacity, respectively. Inactive units are shown in grey with a stop symbol. **b**, Conceptual flow-equalisation strategy under routine 24-h fluctuations. The blue curve represents unadjusted influent flow, the solid black line indicates the design-load threshold, and the solid grey line indicates the equalised flow delivered to treatment. Red-shaded regions denote excess peak flow that would exceed the design-load threshold without equalisation and is instead directed to the influent storage tank; the blue-shaded region denotes the off-peak flow deficit supplemented by releasing stored wastewater. **c**, Application of the two strategies at the Shenzhen Longhua Phase 1 wastewater treatment plant (WWTP) during the 2023 Spring Festival travel rush. The upper plot shows the number of operational treatment units, and the lower plot compares the treatment and inflow dynamic equilibrium (TIDE) index under no intervention (black squares), gradient shutdown only (blue circles), and combined gradient shutdown with flow equalisation (green triangles). The grey bars in the lower plot show the daily population heat value. The blue shading indicates the TIDE gain from gradient shutdown relative to no intervention, whereas the yellow shading indicates the TIDE gain from flow equalisation relative to gradient shutdown alone. **d**, Monthly total GHG emissions from 35 wastewater treatment plants in Shenzhen before (grey bars) and after (blue bars) optimisation from January to June 2023. Black lines represent the relative changes from the no-intervention baseline, with square markers representing TIDE improvement and circular markers representing total GHG emission reduction.

During the Spring Festival travel rush, Shenzhen experiences large-scale outbound migration as residents return to their hometowns (Fig. 2a and b). Domestic wastewater volumes decline steadily with population outflow, reducing the operational efficiency of WWTPs (Fig. 1a, shaded area). We propose a gradient-shutdown strategy in which wastewater treatment units are sequentially shut down as influent volumes decline. For example, in a conceptual treatment plant with three parallel units, the first and second units are shut down when the daily influent volume decreases to two-thirds and one-third of the design capacity, respectively (Fig. 5a).

Under regular daily conditions, influent flows exhibit pronounced peaks and troughs due to the combined effects of intercity commuting, intracity mobility, and water usage habits. Thus, during off-peak hours, treatment facilities operate inefficiently, while during peak hours, they may become overloaded or even experience overflow. We therefore propose a flow equalisation strategy that uses influent storage tanks to retain wastewater during peak hours and then release it during off-peak periods, thereby balancing the flow throughout the day (Fig. 5b).

Previous studies have reported economic and energy benefits of flexible influent load management [9,[46], [47], [48], [49]], but these advantages have not been systematically quantified or translated into generalisable implementation strategies. The main barrier lies in a measurement gap: census-based approaches cannot capture real-time population mobility, making fluctuations in wastewater generation difficult to anticipate. Population heatmap data capture near-real-time relative changes in human distribution and can therefore serve as a proxy for dynamic population presence, improving the accuracy of domestic wastewater prediction under population mobility dynamics. In our analysis, the baseline represents the actual operating conditions of each WWTP. The hourly influent flow, electricity consumption, and chemical consumption used in the analysis were all obtained from real plant operation records. Hence, any effects of existing equalisation facilities or on-site control systems were already reflected in the observed baseline, and the estimated mitigation potential represents the additional improvement achievable by increasing the TIDE under current operating conditions.

We developed influent flow forecasting models for 27 wastewater service areas in Shenzhen using long short-term memory (LSTM) models (Supplementary Text S3). For one-, three-, and 7-h-ahead influent predictions, models incorporating population heatmap data achieved average prediction performance improvements of 1.3%, 10.9%, and 17.9%, respectively, relative to the baseline models. These improvements provide support for the implementation of the two proposed operational strategies.

We then assessed the practical potential of these strategies using Longhua Phase 1 WWTP in Shenzhen as a case study (Supplementary Text S1). During the Spring Festival travel rush, approximately 70% of the wastewater-generating population left the plant's service area, resulting in a 78% reduction in the TIDE. The gradient-shutdown strategy increased TIDE by an average of 5.7%, and the combined use of gradient shutdown and flow equalisation increased TIDE by 18.2% (Fig. 5c). We also conducted an annual-scale assessment of the decarbonisation potential of the two strategies (Fig. 5d). To ensure the cost-effectiveness of the optimisation, only days with a TIDE improvement greater than 20% were selected for scheduling (*n* = 60, 24.7% of rain-free days; see Supplementary Text S4 for the rationale). The results show an average annual reduction in GHG emissions of 17.5%, with the largest decreases occurring in January and February, coinciding with the Spring Festival travel rush.

These strategies and estimated mitigation benefits represent theoretical optimisation scenarios rather than directly validated full-scale operating outcomes. Practical implementation will require plant-level verification of the storage capacity, pumping configuration, process layout, operational stability, recovery time, effluent compliance, odour control, and auxiliary control measures. Therefore, the reported mitigation potential should be interpreted as an estimate of achievable mitigation under improved treatment–inflow alignment, rather than as an immediately transferable operational result.

### 3.6 Quantifying the decarbonisation potential from the plant to the national scale

We extended our analysis to the national scale for 2021–2023 (Supplementary Fig. S4). City-level migration intensity, defined as the difference between the inflow index and the outflow index on Chinese New Year's Eve in each year (February 11, 2021; January 31, 2022; and January 21, 2023), reveals widespread intercity mobility during the Spring Festival travel rush, particularly east of the Hu Huanyong Line, which separates densely populated eastern China from sparsely populated western China (Supplementary Fig. S4a). Provincial capitals and major urban agglomerations, including the Yangtze River Delta and the Greater Bay Area, generally showed net outflows as non-local migrant workers returned to their hometowns, whereas other areas exhibited net inflows as local residents who had worked elsewhere returned. These migration flows have intensified since the end of the COVID-19 pandemic, likely reflecting the relaxation of mobility restrictions and the recovery of intercity and employment-related travel (Supplementary Fig. S5). Such population movements are not unique to China but are observed in countries worldwide; for example, during Christmas in the United States, Diwali in India, Songkran in Thailand, and Lunar New Year (Tết) in Vietnam.

Using a top-down approach (Section 2.2), we evaluated TIDE across Chinese cities. Consistent with Shenzhen's results, the conventional load rate metrics overestimated the treatment–inflow alignment at the national level, with an average overestimation of 15.9% (Fig. 1a; Supplementary Fig. S6). While nationwide monitoring and process data were unavailable to directly quantify the GHG mitigation potential, city-level TIDE-optimisation potential was used as an indirect indicator of potential mitigation opportunities.

From 2021 to 2023, the national average TIDE increased from 0.63 to 0.64. Higher TIDE values were more frequently observed in northern Chinese cities, although the mechanism may differ across regions. In some northwestern and northeastern cities, higher TIDE values may be related to a combination of lower population density, weaker population mobility, more stable domestic wastewater inflow, and less pronounced rainfall-driven infiltration and inflow. In northern cities with larger populations, higher TIDE values may also partly reflect a relatively stable baseline influent load that dilutes mobility-driven fluctuations, such as wastewater from industrial or production-related activities. In contrast, many southern cities had lower TIDE values but higher ΔTIDE (defined as the theoretical increase in TIDE achieved after applying the two proposed strategies), indicating greater potential to improve treatment–inflow alignment through operational optimisation (Supplementary Fig. S4c). This may be associated with stronger population mobility, faster urbanisation, and greater rainfall-driven infiltration and inflow, which can amplify the temporal mismatches between dynamic wastewater inflow and fixed treatment capacity.

Applying the proposed strategies yielded an average theoretical TIDE improvement of 22.2% across cities (Supplementary Fig. S4c), indicating widespread potential for improving treatment–inflow alignment in China. TIDE and ΔTIDE can serve as useful diagnostic indicators for identifying which governance pathway may be most suitable for different cities, while the annual load rate can help diagnose the source of any mismatch. Cities with high TIDE and limited ΔTIDE should focus on maintaining the existing alignment, avoiding unnecessary capacity expansion, and improving process-level energy efficiency. Cities with low TIDE but high ΔTIDE are more suitable venues for operational strategies such as flow equalisation and mobility-informed scheduling, especially when the daily or holiday-related inflow fluctuations are strong. Furthermore, cities with low TIDE, low ΔTIDE, and low load rates may require sewer network improvements, service area verification, or capacity planning corrections, whereas cities with low TIDE, low ΔTIDE, and persistently high load rates may require infiltration and inflow control, infrastructure upgrades, or treatment capacity expansion.

## 4 Conclusions

Spatiotemporal mismatches between WWTP capacity and dynamic population distributions challenge sustainable wastewater management because wastewater generation varies with activities such as commuting, schooling, medical visits and tourism—dynamics that are not adequately captured by static statistics. To address this gap, we integrated Baidu population heatmap data as a proxy for dynamic population presence and developed TIDE to evaluate treatment–inflow alignment. Conventional load rates were found to overestimate this alignment by overlooking spatiotemporal variations in wastewater generation. In Shenzhen, population outflow during the Spring Festival travel rush was associated with lower TIDE and higher GHG emission intensity, whereas intercity commuting was associated with improved treatment–inflow alignment and lower estimated GHG emissions. Together, these findings indicate that dynamic population information may support more responsive WWTP management.

Several uncertainties remain. First, although direct CH_4_ and N_2_O emissions were included in the GHG accounting framework, potential changes in these emissions induced by TIDE-based optimisation were not included in the estimated mitigation benefit. This is because high-frequency process emissions and operational data were unavailable. Such operational changes may affect hydraulic retention time, dissolved oxygen, and organic carbon availability, thereby influencing direct CH_4_ and N_2_O emissions. Second, population heatmap data indicate relative rather than absolute population presence and may underrepresent activity in scenic, mountainous, or remote areas, as well as during late-night periods with sparse location-service signals. Moreover, population data alone cannot capture other drivers of influent flow, including industrial inputs, infiltration and inflow, rainfall, and water-use behaviour. Third, the estimated operational optimisation potential represents a theoretical maximum that may not be fully achievable under existing plant constraints. Future studies should integrate high-frequency operational and process-emission data and validate the achievable benefits under plant-specific conditions.

Beyond operational optimisation, TIDE may also provide a complementary indicator for WWTP planning and design. In areas characterised by pronounced seasonal or commuting-related population shifts and uncertain future growth, WWTP design should not rely solely on a fixed capacity based on static population statistics; it should also account for variations in actual influent load. TIDE may help identify where flexible operating configurations, modular treatment, or phased capacity expansion could improve alignment between treatment capacity and variable influent loads.

With the widespread use of smart mobile devices and location-based services, mobile location data offer growing opportunities to characterise dynamic population distributions. Population movements during holidays (e.g., the Spring Festival and Christmas) and interdistrict or intercity commuting are common in many regions, suggesting that the proposed framework may be transferable to settings with comparable population mobility and WWTP operational data. In regions with low-carbon electricity systems such as Norway and Iceland, its benefits may be reflected primarily in energy and chemical savings and operational stability, whereas GHG mitigation should be evaluated using local electricity mixes and emission factors. Overall, integrating dynamic population information into WWTP operational decisions can help operators adjust treatment-unit operation and influent allocation in response to shifting wastewater loads, thereby improving treatment–inflow alignment and reducing treatment-related GHG emissions.

## CRediT authorship contribution statement

**Yiming Meng:** Writing – original draft, Investigation, Formal analysis, Conceptualization. **Yu Tian:** Project administration, Methodology, Funding acquisition, Conceptualization. **Yu Tao:** Project administration, Methodology, Conceptualization. **Shupeng Wang:** Investigation, Formal analysis. **Purui Wang:** Visualization, Data curation. **Mengxin Li:** Visualization, Data curation. **Lingchao Kong:** Writing – review & editing. **Andy Deng:** Writing – review & editing. **Huihang Sun:** Writing – review & editing. **Wei Zhan:** Investigation, Data curation. **Jiang Yu:** Investigation, Data curation. **Xindan Fan:** Investigation, Data curation. **Yong Tian:** Supervision. **Chunmiao Zheng:** Supervision.

## Data availability

The raw population mobility data used in this study were provided by Baidu Huiyan. These data can be requested by contacting their service team via https://huiyan.baidu.com/. Anonymised, grid-aggregated datasets used to reproduce our findings will be made available upon reasonable request.

Data on wastewater treatment capacity, total treated volume, and the number of WWTPs in Chinese cities were sourced from the *China Urban Construction Statistical Yearbook*. Data on GHG emissions from WWTPs—including electricity use, chemical consumption, and external carbon sources—were obtained from the National Urban Wastewater Treatment Management Information System. Both resources are released by the Ministry of Housing and Urban-Rural Development of the People's Republic of China and are available through http://www.mohurd.gov.cn.

Influent flow data of 35 WWTPs in Shenzhen were obtained from the Water Authority of Shenzhen Municipality. A strict confidentiality agreement was established between both parties to ensure that the data are used exclusively for this study and are therefore not publicly available. Interested parties may submit access requests in accordance with the Shenzhen Water Authority's Information Disclosure Guidelines via https://swj.sz.gov.cn/. The Shenzhen WWTP dataset includes plant-level design capacities and core treatment processes (Supplementary Table S4).

## Declaration of competing interests

The authors declare that they have no known competing financial interests or personal relationships that could have appeared to influence the work reported in this paper. Dr. Yu Tao, the Managing Editor of Environmental Science and Ecotechnology, was not involved in the editorial review or the decision to publish this article.

## References

[1] Rahman M.F. (2023). As the UN meets, make water central to climate action. Nature.

[2] Khanna N., Lin J., Liu X., Wang W. (2024). An assessment of China's methane mitigation potential and costs and uncertainties through 2060. Nat. Commun..

[3] Du W.J. (2023). Spatiotemporal pattern of greenhouse gas emissions in China's wastewater sector and pathways towards carbon neutrality. Nat. Water.

[4] Chen S. (2023). Decoupling wastewater-related greenhouse gas emissions and water stress alleviation across 300 cities in China is challenging yet plausible by 2030. Nat. Water.

[5] He Z. (2025). Water conservation strategies reduce greenhouse gas emission from wastewater treatment plants: a domino effect. Environ. Sci. Ecotechnol..

[6] International Energy Agency (2017).

[7] State Statistics Bureau (2023).

[8] Simon-Varhelyi M., Cristea V.M., Luca A.V. (2020). Reducing energy costs of the wastewater treatment plant by improved scheduling of the periodic influent load. J. Environ. Manag..

[9] Rao A.K., Bolorinos J., Musabandesu E., Chapin F.T., Mauter M.S. (2024). Valuing energy flexibility from water systems. Nat. Water.

[10] Yang J., Chen B. (2021). Energy efficiency evaluation of wastewater treatment plants (WWTPs) based on data envelopment analysis. Appl. Energy.

[11] Gao K., Yang H., Zhao Q., Liu H. (2023). Identification of key basic parameters involved in carbon emissions in full-scale wastewater treatment plants. Sustainability.

[12] Niu K., Wu J., Qi L., Niu Q. (2019). Energy intensity of wastewater treatment plants and influencing factors in China. Sci. Total Environ..

[13] Reifsnyder S., Cecconi F., Rosso D. (2021). Dynamic load shifting for the abatement of GHG emissions, power demand, energy use, and costs in metropolitan hybrid wastewater treatment systems. Water Res..

[14] Dai W. (2025). Integrated real-time intelligent control for wastewater treatment plants: Data-driven modeling for enhanced prediction and regulatory strategies. Water Res..

[15] De La Hoz J.L.M., Bappy M.M., Islam M.S., Marcantel M., Hayes M.P. (2026). Interpretable forecasting of dissolved oxygen leveraging foundation model for proactive aeration in rural wastewater treatment systems. Water Res..

[16] Yin Y., Wu Q., Li M. (2023). Characterizing intercity mobility patterns for the greater Bay area in China. ISPRS Int. J. GeoInf..

[17] The People's Government of Guangzhou Municipality (2025). Approximately 1.29 million cross city commuters in the nine cities of the Greater Bay Area in 2024. https://www.gz.gov.cn/zt/qltjygadwqjsxsdzgzlfzdf/mtjj/content/post_10393470.html.

[18] Ministry of Transport of the People's Republic of China (2023). The Spring Festival Travel 2023 concluded successfully, with more than 4.7 billion trips made. https://www.mot.gov.cn/zhuanti/2023chunyun/chunyundongtai/202302/t20230216_3758346.html.

[19] Lai S. (2022). Global holiday datasets for understanding seasonal human mobility and population dynamics. Sci. Data.

[20] Motamed B., Shirvanimoghaddam K. (2021). The local Co-Working hub: a merging solution. Urban Sci..

[21] Zhou K., Wu J., Liu H. (2021). Spatiotemporal variations and determinants of water pollutant discharge in the Yangtze River Economic Belt, China: a spatial econometric analysis. Environ. Pollut..

[22] Ding L., Lv Z., Han M., Zhao X., Wang W. (2019). Forecasting China's wastewater discharge using dynamic factors and mixed-frequency data. Environ. Pollut..

[23] Liu B., Wang S., Tang Y., Yan B. (2023). Prediction of wastewater discharge based on GRA-LSTM: a case study of Beijing. Environ. Sci. Pollut. Res..

[24] Batista e Silva F. (2020). Uncovering temporal changes in Europe's population density patterns using a data fusion approach. Nat. Commun..

[25] Nilforoshan H. (2023). Human mobility networks reveal increased segregation in large cities. Nature.

[26] Tan X. (2025). The spatiotemporal scaling laws of urban population dynamics. Nat. Commun..

[27] Hong B., Bonczak B.J., Gupta A., Kontokosta C.E. (2021). Measuring inequality in community resilience to natural disasters using large-scale mobility data. Nat. Commun..

[28] Moro E., Calacci D., Dong X., Pentland A. (2021). Mobility patterns are associated with experienced income segregation in large US cities. Nat. Commun..

[29] Abbiasov T. (2024). The 15-minute city quantified using human mobility data. Nat. Hum. Behav..

[30] Nyhan M. (2016). "Exposure track"—the impact of mobile-device-based mobility patterns on quantifying population exposure to air pollution. Environ. Sci. Technol..

[31] Testi I. (2024). Big mobility data reveals hyperlocal air pollution exposure disparities in the Bronx, New York. Nature Cities.

[32] Yan X. (2024). Spatiotemporal infection dynamics: linking individual movement patterns to infection status. Cities.

[33] Tran-Kiem C. (2025). Fine-scale patterns of SARS-CoV-2 spread from identical pathogen sequences. Nature.

[34] Kostandova N. (2024). A systematic review of using population-level human mobility data to understand SARS-CoV-2 transmission. Nat. Commun..

[35] Grantz K.H. (2020). The use of mobile phone data to inform analysis of COVID-19 pandemic epidemiology. Nat. Commun..

[36] Rajput A.A., Liu C., Liu Z., Mostafavi A. (2024). Human-centric characterization of life activity flood exposure shifts focus from places to people. Nature Cities.

[37] Gibbs H. (2020). Changing travel patterns in China during the early stages of the COVID-19 pandemic. Nat. Commun..

[38] Zhang Q.H. (2016). Current status of urban wastewater treatment plants in China. Environ. Int..

[39] The Intergovernmental Panel on Climate Change (2014).

[40] China Association of Environmental Protection Industry (2022).

[41] Zhou Z., Li H.B., Wang Y., Li J. (2022). Research on carbon emission characteristics and low-carbon operation mode in AAO-based wastewater treatment plants with low C/N influent. China Environ. Sci..

[42] Statistics Bureau of Shenzhen Municipality (2025).

[43] Water Authority of Shenzhen Municipality (2024). How many river basins can be divided in our city?. https://swj.sz.gov.cn/gzhd/ywzsk/hd/content/post_11693509.html.

[44] Qiu N. (2025). Active commuting: an alternative source of health-promoting physical activity in job-housing imbalance contexts. Hum. Soc. Sci. Commun..

[45] Zhao J., Mo B., Caros N.S., Zhao J. (2025). Housing exchange framework to reduce carbon emissions from commuting. Nat. Sustain..

[46] Technical Centre for Soil (2024). Agriculture and rural ecology and environment, ministry of ecology and environment. Adapting measures to local conditions to control tides, striving for practical results, and overcoming difficulties—Typical models and cases of "tidal" rural domestic sewage treatment.

[47] Bolorinos J., Mauter M.S., Rajagopal R. (2023). Integrated energy flexibility management at wastewater treatment facilities. Environ. Sci. Technol..

[48] Chapin F.T., Wettermark D., Bolorinos J., Mauter M.S. (2025). Load-shifting strategies for cost-effective emission reductions at wastewater facilities. Environ. Sci. Technol..

[49] Carucci A., Rolle E., Smurra P. (1999). Management optimisation of a large wastewater treatment plant. Water Sci. Technol..

