# Supplementary material for: Human mobility acts as a lever for decarbonising wastewater treatment plants
Source: Environ Sci Ecotechnol. 2026 Aug 20;33:100756. doi: 10.1016/j.ese.2026.100756 (PMC13545441; doi:10.1016/j.ese.2026.100756)
Supplement: Supplementary file 1 [file mmc1.docx]

**Supplementary Materials for**

**Human Mobility Acts as a Lever for Decarbonising Wastewater Treatment Plants**

Yiming Meng^1^, Yu Tian^1, *^, Yu Tao^2^, Shupeng Wang^1^, Purui Wang^1^, Mengxin Li^1^,

Lingchao Kong^3, 4^, Andy Deng^5^, Huihang Sun^1^, Wei Zhan^1^, Jiang Yu^4^, Xindan Fan^6^, Yong Tian^4, *^, and Chunmiao Zheng^3, 4^

^1^ State Key Laboratory of Urban-rural Water Resource and Environment, School of Environment, Harbin Institute of Technology, Harbin 150090, China

^2^ School of Civil and Environmental Engineering, Harbin Institute of Technology (Shenzhen), Shenzhen 518055, China

^3^ School of the Environment and Sustainable Engineering, Eastern Institute of Technology, Ningbo 315200, China

^4^ State Key Laboratory of Soil Pollution Control and Safety, School of Environmental Science and Engineering, Southern University of Science and Technology, Shenzhen 518055, China

^5^ School of Mechanical and Manufacturing Engineering, University of New South Wales, Sydney NSW 2052, Australia

^6^ Guanlan River Basin Management Center, Water Authority of Shenzhen Municipality, Shenzhen 518110, China

*Corresponding author: tianyu@hit.edu.cn (Yu Tian); tiany@sustech.edu.cn (Yong Tian)

**Summary Information**

Number of pages: 30

Tables: S1–S10

Figures: S1–S13

Texts: S1-S6

Supplementary Tables:

**Supplementary Table 1. Hourly Discharge Profiles by Zone**

| HOUR | Zone I^1,2^ | Zone II^3,4^ | Zone III^5,6^ | Zone IV^7,8^ | Zone V^9,10^ | Zone VI^11,12^ |
| --- | --- | --- | --- | --- | --- | --- |
| 0 | 3.44% | 2.58% | 3.54% | 2.40% | 4.71% | 3.96% |
| 1 | 2.39% | 1.86% | 2.65% | 1.95% | 2.92% | 2.54% |
| 2 | 1.44% | 1.38% | 1.80% | 1.39% | 1.74% | 0.87% |
| 3 | 1.14% | 1.25% | 1.54% | 1.15% | 1.28% | 0.47% |
| 4 | 1.04% | 1.10% | 1.46% | 1.11% | 1.08% | 0.43% |
| 5 | 1.13% | 1.11% | 1.55% | 1.10% | 1.16% | 0.74% |
| 6 | 3.47% | 3.76% | 6.75% | 2.56% | 2.23% | 2.04% |
| 7 | 4.97% | 4.33% | 6.24% | 5.50% | 4.42% | 4.36% |
| 8 | 5.46% | 5.03% | 6.24% | 5.47% | 5.16% | 5.38% |
| 9 | 5.33% | 5.27% | 6.24% | 5.62% | 5.03% | 4.50% |
| 10 | 5.22% | 5.62% | 6.24% | 5.31% | 4.73% | 4.20% |
| 11 | 5.64% | 5.32% | 6.30% | 6.47% | 4.58% | 5.51% |
| 12 | 5.32% | 5.34% | 3.97% | 6.08% | 4.41% | 6.81% |
| 13 | 4.80% | 5.65% | 3.97% | 3.84% | 3.97% | 6.58% |
| 14 | 4.27% | 4.64% | 3.97% | 3.71% | 3.33% | 5.67% |
| 15 | 3.75% | 4.30% | 3.97% | 3.83% | 3.08% | 3.58% |
| 16 | 3.76% | 4.49% | 3.97% | 3.95% | 3.13% | 3.27% |
| 17 | 4.07% | 4.71% | 3.97% | 4.06% | 3.64% | 3.97% |
| 18 | 4.73% | 5.08% | 3.92% | 4.52% | 4.63% | 5.52% |
| 19 | 5.89% | 5.22% | 3.80% | 7.77% | 5.64% | 7.03% |
| 20 | 6.06% | 5.81% | 3.80% | 7.42% | 6.29% | 6.98% |
| 21 | 6.07% | 6.16% | 3.80% | 6.58% | 7.81% | 5.99% |
| 22 | 5.90% | 5.30% | 5.63% | 4.98% | 8.20% | 5.37% |
| 23 | 4.71% | 4.67% | 4.64% | 3.21% | 6.82% | 4.23% |

Note: Zoning is based on *the Handbook of Calculation Methods and Coefficients for Emission Source Statistics and Investigation* from the Ministry of Ecology and Environment of the People's Republic of China^13^. Mainland China is divided into six zones according to administrative divisions, geography, climate, economy, and water usage patterns.

**Supplementary Table 2. Emission factors of chemicals**

| Chemicals | $EF_{CO_{2}}$（kg CO_2_/kg） |
| --- | --- |
| CH_3_COONa | 1.6 |
| CH_3_OH | 1.54 |
| Other external carbon source | 1.6 |
| Poly Aluminium Chloride | 1.62 |
| Polyferric Sulfate | 2.5 |
| Other phosphorus removing agent | 2.5 |
| Polyacrylamide | 1.5 |
| Other dewatering agent source | 1.6 |

Source: Parravicini V etal^14^.

**Supplementary Table 3. Material Consumption–TIDE Regression Results for Shenzhen’s WWTPs**

| No. | Plant | Material | Chemical | Equation | R^2^ | DataN |
| --- | --- | --- | --- | --- | --- | --- |
| 1 | Banxuegang WWTP (Phase 2) | Electricity | -- | y = -0.8022x + 1.1112 | 0.917 | 33 |
|  |  | Carbon Source | Sodium Acetate | y = 10817e^-6.064x^ | 0.6057 | 14 |
|  |  | Phosphorus Agent | Polyaluminum Chloride | y = 10305e^-3.3x^ | 0.655 | 27 |
|  |  | Sludge Agent | Polyacrylamide | y = -6.9881x + 10.887 | 0.6267 | 30 |
| 2 | Banxuegang WWTP (Phase 1) | Electricity | -- | y = -0.303ln(x) + 0.2809 | 0.669 | 19 |
|  |  | Phosphorus Agent | Polyaluminum Chloride | y = -753ln(x) + 207.74 | 0.1936 | 24 |
|  |  | Sludge Agent | Polyacrylamide | y = -3.128ln(x) + 8.705 | 0.1911 | 15 |
| 3 | Binhe WWTP | Electricity | -- | y = -0.5662x + 1.0567 | 0.6855 | 29 |
| 4 | Buji WWTP  (Phase 1) | Electricity | -- | y = 1.0714e^-1.506x^ | 0.7228 | 32 |
|  |  | Phosphorus Agent | Polyaluminum Chloride | y = 21986e^-7.084x^ | 0.4858 | 14 |
|  |  | Sludge Agent | Polyacrylamide | y = -18.511x + 21.096 | 0.4398 | 31 |
| 5 | Egongling WWTP | Electricity | -- | y = -0.4978x + 0.7769 | 0.7057 | 19 |
|  |  | Carbon Source | Sodium Acetate | y = 1677.5e^-2.517x^ | 0.155 | 19 |
|  |  | Phosphorus Agent | Polyaluminum Chloride | y = 2901.7e^-1.759x^ | 0.5011 | 16 |
|  |  | Sludge Agent | Polyacrylamide | y = 60.889e^-3.626x^ | 0.6779 | 18 |
| 6 | Futian WWTP | Electricity | -- | y = -0.4391x + 0.7475 | 0.6454 | 16 |
|  |  | Phosphorus Agent | Polyaluminum Chloride | y = -861.23x + 1046 | 0.4278 | 13 |
|  |  | Sludge Agent | Polyacrylamide | y = 13.585e^-1.23x^ | 0.0489 | 20 |
| 7 | Fuyong WWTP | Electricity | -- | y = -0.2823x + 0.6302 | 0.6593 | 22 |
|  |  | Phosphorus Agent | Others | y = 3158.1e^-1.483x^ | 0.5416 | 15 |
|  |  | Sludge Agent | Polyacrylamide | y = -0.929ln(x) + 4.2545 | 0.0042 | 21 |
| 8 | Gongming WWTP | Electricity | -- | y = -0.2174x + 0.5686 | 0.4671 | 18 |
|  |  | Phosphorus Agent | Polyaluminum Chloride | y = 9471.1e^-2.662x^ | 0.5162 | 22 |
|  |  | Sludge Agent | Polyacrylamide | y = 83.715e^-2.672x^ | 0.5635 | 22 |
| 9 | Gushu WWTP  (Phase 1) | Electricity | -- | y = -0.2217x + 0.5403 | 0.5519 | 34 |
|  |  | Carbon Source | Sodium Acetate | y = -481.34x + 599.47 | 0.4398 | 21 |
|  |  | Phosphorus Agent | Others | y = -802.63x + 1579.9 | 0.444 | 25 |
|  |  | Sludge Agent | Polyacrylamide | y = -8.5962x + 16.251 | 0.4823 | 19 |
| 10 | Guanlan WWTP  (Phase 2) | Electricity | -- | y = -0.6818x + 0.8759 | 0.8148 | 21 |
|  |  | Carbon Source | Sodium Acetate | y = 107.29 | -- | 17 |
|  |  | Phosphorus Agent | Others | y = -2309.8x + 2482.9 | 0.4105 | 12 |
| 11 | Guanlan WWTP  (Phase 1) | Electricity | -- | y = -0.3254x + 0.5435 | 0.685 | 21 |
| 12 | Guangming WWTP | Electricity | -- | y = -0.2596x + 0.5706 | 0.8033 | 25 |
|  |  | Carbon Source | Sodium Acetate | y = 492644e^-9.472x^ | 0.4583 | 18 |
|  |  | Phosphorus Agent | Polyaluminum Chloride | y = -915.52x + 1693.3 | 0.5128 | 12 |
|  |  | Sludge Agent | Polyacrylamide | y = 1.12 | -- | 25 |
| 13 | Henggang WWTP (Phase 2) | Electricity | -- | y = 2.4839e^-3.233x^ | 0.628 | 13 |
|  |  | Carbon Source | Others | y = 6575.1e^-3.977x^ | 0.466 | 15 |
|  |  | Phosphorus Agent | Polyaluminum Chloride | y = 794.7e^-0.474x^ | 0.0109 | 20 |
| 14 | Henggang WWTP (Phase 1) | Electricity | -- | y = -0.178ln(x) + 0.2201 | 0.8784 | 32 |
|  |  | Carbon Source | Sodium Acetate | y = 614.47e^-0.235x^ | 0.0015 | 28 |
|  |  | Phosphorus Agent | Polyaluminum Chloride | y = 412.84 | -- | 21 |
|  |  | Sludge Agent | Polyacrylamide | y = -9.8312x + 13.106 | 0.7162 | 18 |
| 15 | Hengling WWTP (Phase 2) | Electricity | -- | y = -0.3072x + 0.5725 | 0.7187 | 25 |
|  |  | Carbon Source | Sodium Acetate | y = 12578e^-2.704x^ | 0.5133 | 13 |
|  |  | Phosphorus Agent | Polyaluminum Chloride | y = -2762.9x + 4677.9 | 0.545 | 22 |
|  |  | Sludge Agent | Polyacrylamide | y = -14.19x + 26.298 | 0.4999 | 23 |
| 16 | Hengling WWTP (Phase 1) | Electricity | -- | y = -0.2083x + 0.4989 | 0.4242 | 34 |
|  |  | Phosphorus Agent | Polyaluminum Chloride | y = -137.01x + 326.31 | 0.5014 | 14 |
|  |  | Sludge Agent | Polyacrylamide | y = 3.9 | -- | 34 |
| 17 | Kuichong WWTP | Electricity | -- | y = -0.2558x + 0.353 | 0.6649 | 23 |
|  |  | Phosphorus Agent | Polyaluminum Chloride | y = 15267e^-7.351x^ | 0.4508 | 17 |
|  |  | Sludge Agent | Polyacrylamide | y = 20.881e^-3.238^ | 0.5738 | 18 |
| 18 | Longhua WWTP  (Phase 2) | Electricity | -- | y = -0.4511x + 0.7644 | 0.7932 | 25 |
|  |  | Phosphorus Agent | Polyaluminum Chloride | y = 2338.5e^-2.336x^ | 0.5672 | 20 |
| 19 | Longhua WWTP  (Phase 1) | Electricity | -- | y = 0.8961e^-1.179x^ | 0.4739 | 14 |
|  |  | Phosphorus Agent | Polyaluminum Chloride | y = -884.52x + 1261.7 | 0.0945 | 15 |
|  |  | Sludge Agent | Polyacrylamide | y = -9.013ln(x) + 3.3064 | 0.4693 | 15 |
| 20 | Longtian WWTP | Electricity | -- | y = -0.418x + 0.5811 | 0.8083 | 28 |
|  |  | Phosphorus Agent | Polyaluminum Chloride | y = 1528.5e^-1.64x^ | 0.5078 | 27 |
|  |  | Sludge Agent | Polyacrylamide | y = -1.0167x + 3.586 | 0.0584 | 20 |
| 21 | Luofang WWTP | Electricity | -- | y = -0.6041x + 0.9905 | 0.6221 | 33 |
|  |  | Phosphorus Agent | Polyaluminum Chloride | y = -1342.3x + 1752.8 | 0.407 | 25 |
|  |  | Sludge Agent | Others | y = 4570.6e^-2.071x^ | 0.5446 | 34 |
| 22 | Nanshan WWTP | Electricity | -- | y = -0.1351x + 0.4769 | 0.6333 | 34 |
|  |  | Phosphorus Agent | Polyaluminum Chloride | y = -200.32x + 501.66 | 0.4538 | 30 |
| 23 | Pinghu WWTP | Electricity | -- | y = -0.3647x + 0.6577 | 0.5512 | 26 |
|  |  | Phosphorus Agent | Polyaluminum Chloride | y = -849.26x + 1455.7 | 0.4716 | 22 |
|  |  | Sludge Agent | Polyacrylamide | y = -7.4565x + 9.4858 | 0.5085 | 19 |
| 24 | Pudixia WWTP  (Phase 2) | Electricity | -- | y = -0.5894x + 0.9007 | 0.5279 | 31 |
|  |  | Carbon Source | Others | y = -12.759x + 114.81 | 0.0002 | 25 |
|  |  | Phosphorus Agent | Polyferric Sulfate | y = 13362e^-4.433x^ | 0.4044 | 27 |
|  |  | Sludge Agent | Others | y = 84.992e^-0.012x^ | 0.0772 | 28 |
| 25 | Pudixia WWTP  (Phase 1) | Electricity | -- | y = -0.3673x + 0.626 | 0.6156 | 22 |
|  |  | Phosphorus Agent | Polyaluminum Chloride | y = 6989.5e^-2.607x^ | 0.5059 | 14 |
|  |  | Sludge Agent | Polyacrylamide | y = -6.8032x + 7.8369 | 0.4469 | 17 |
| 26 | Shajing WWTP  (Phase 2) | Electricity | -- | y = -0.2742x + 0.5298 | 0.8712 | 32 |
|  |  | Carbon Source | Sodium Acetate | y = 107783e^-10.24x^ | 0.504 | 14 |
|  |  | Phosphorus Agent | Polyferric Sulfate | y = 467.09 | -- | 28 |
|  |  | Sludge Agent | Polyacrylamide | y = -7.9828x + 11.864 | 0.5719 | 23 |
| 27 | Shajing WWTP  (Phase 1) | Electricity | -- | y = -0.3859x + 0.5855 | 0.8231 | 24 |
|  |  | Carbon Source | Sodium Acetate | y = 7497.3e^-1.888x^ | 0.6127 | 16 |
|  |  | Phosphorus Agent | Polyaluminum Chloride | y = -263.8ln(x) + 954.83 | 0.0906 | 15 |
|  |  | Sludge Agent | Polyacrylamide | y = 93.804e^-2.01x^ | 0.7788 | 17 |
| 28 | Shatian WWTP | Electricity | -- | y = -0.401x + 0.7231 | 0.5492 | 26 |
|  |  | Phosphorus Agent | Polyaluminum Chloride | y = -621.4ln(x) + 355 | 0.6242 | 21 |
|  |  | Sludge Agent | Polyacrylamide | y = -4.795ln(x) + 1.2109 | 0.6516 | 23 |
| 29 | Shangyang WWTP | Electricity | -- | y = -0.1398x + 0.4121 | 0.884 | 33 |
|  |  | Phosphorus Agent | Polyaluminum Chloride | y = -389.14x + 546.8 | 0.4256 | 27 |
| 30 | Shekou WWTP | Electricity | -- | y = -0.2604x + 0.7223 | 0.6895 | 34 |
|  |  | Phosphorus Agent | Polyaluminum Chloride | y = -714.6x + 1305.1 | 0.4615 | 27 |
| 31 | Shuitou WWTP | Electricity | -- | y = -0.119ln(x) + 0.2134 | 0.4592 | 16 |
|  |  | Phosphorus Agent | Polyaluminum Chloride | y = 375722e^-12.68x^ | 0.4045 | 12 |
| 32 | Songgang WWTP (Phase 2) | Electricity | -- | y = -0.3211x + 0.5464 | 0.5897 | 19 |
|  |  | Phosphorus Agent | Polyferric Sulfate | y = 557.89 | -- | 20 |
|  |  | Sludge Agent | Polyacrylamide | y = -4.667ln(x) + 1.8828 | 0.4937 | 18 |
| 33 | Songgang WWTP (Phase 1) | Electricity | -- | y = -0.389x + 0.6904 | 0.5297 | 17 |
|  |  | Phosphorus Agent | Others | y = -1567.3x + 2017 | 0.42 | 13 |
|  |  | Sludge Agent | Polyacrylamide | y = -10.53x + 13.082 | 0.4809 | 15 |
| 34 | Xili WWTP | Electricity | -- | y = -0.7567x + 0.9054 | 0.6634 | 26 |
|  |  | Carbon Source | Methanol | y = 4527.3e^-3.747x^ | 0.6536 | 25 |
|  |  | Phosphorus Agent | Polyaluminum Chloride | y = -1779ln(x) + 562.22 | 0.6231 | 24 |
|  |  | Sludge Agent | Polyacrylamide | y = 35.923e^-2.702x^ | 0.6146 | 23 |
| 35 | Yantian WWTP | Electricity | -- | y = -0.4679x + 0.6887 | 0.7449 | 34 |
|  |  | Phosphorus Agent | Polyaluminum Chloride | y = -1004ln(x) + 399.54 | 0.6794 | 33 |
|  |  | Sludge Agent | Polyacrylamide | y = 16.33e^-1.712x^ | 0.6454 | 28 |

Source: National Urban Wastewater Treatment Management Information System, https://wushui.mohurd.gov.cn:8088/login.jsp.

Note: Fitting results are based on monthly averages of TIDE and reported material consumption at each plant. Due to data limitations, only available and analyzable records are presented. For each material type, the final regression equation was selected by comparing multiple functional forms and choosing the one with the highest coefficient of determination (R²).

**Supplementary Table 4. Design parameters for WWTPs**

| No. | Plant name | Core Process | Design Capacity  (10⁴ m³/day) |
| --- | --- | --- | --- |
| 1 | Banxuegang WWTP (Phase 1) | Modified A^2^O | 4 |
| 2 | Banxuegang WWTP (Phase 2) | A^2^O | 12 |
| 3 | Binhe WWTP | AB | 30 |
| 4 | Buji WWTP (Phase 1) | Modified A^2^O | 20 |
| 5 | Egongling WWTP | Modified A^2^O | 5 |
| 6 | Futian WWTP | Modified A^2^O | 5 |
| 7 | Fuyong WWTP | Modified A^2^O | 12.5 |
| 8 | Gongming WWTP | Modified A^2^O | 10 |
| 9 | Guangming WWTP | Modified A^2^O | 30 |
| 10 | Guanlan WWTP (Phase 1) | Tertiary A/O | 16 |
| 11 | Guanlan WWTP (Phase 2) | Modified A^2^O | 24 |
| 12 | Gushu WWTP (Phase 1) | Modified A^2^O | 24 |
| 13 | Henggang WWTP (Phase 1) | SBR | 10 |
| 14 | Henggang WWTP (Phase 2) | Modified A^2^O | 10 |
| 15 | Hengling WWTP (Phase 1) | UTC | 20 |
| 16 | Hengling WWTP (Phase 2) | Aerobic Biofilter | 40 |
| 17 | Kuichong WWTP | CASS | 4 |
| 18 | Longhua WWTP (Phase 1) | MBBR | 15 |
| 19 | Longhua WWTP (Phase 2) | Modified A^2^O | 25 |
| 20 | Longtian WWTP | A^2^O | 15 |
| 21 | Luofang WWTP | BNR + MBR | 8 |
| 22 | Nanshan WWTP | MUCT | 56 |
| 23 | Pinghu WWTP | Modified A^2^O | 8 |
| 24 | Pudixia WWTP (Phase 1) | A^2^O | 5 |
| 25 | Pudixia WWTP (Phase 2) | Multi-stage A/O | 5 |
| 26 | Shajing WWTP (Phase 1) | A^2^O | 15 |
| 27 | Shajing WWTP (Phase 2) | A^2^O | 35 |
| 28 | Shangyang WWTP | A^2^O | 20 |
| 29 | Shatian WWTP | A^2^O | 3 |
| 30 | Shekou WWTP | CASS | 5 |
| 31 | Shuitou WWTP | CASS | 4 |
| 32 | Songgang WWTP (Phase 1) | Modified A^2^O | 15 |
| 33 | Songgang WWTP (Phase 2) | MBBR | 15 |
| 34 | Xili WWTP | BAF | 5 |
| 35 | Yantian WWTP | A^2^O | 12 |

Source: Shenzhen Water Authority (https://swj.sz.gov.cn/).

Supplementary Figures:

**Supplementary Figure 1. Construction progress of wastewater treatment plants in China (2021–2023).** **a** Annual domestic wastewater discharge intensity at the city level, measured as total domestic wastewater volume per unit area. **b** Number of operational WWTPs in each city. Notably, this number is limited to the statistical figure in the building sector. **c** Total daily design capacity of WWTPs per city. **d** Wastewater load rate per city, calculated as the ratio of daily influent volume to design capacity (see Methods for details). Values greater than 1 (red areas) indicate that the existing treatment capacity is insufficient to meet the city’s wastewater treatment demand.

**Supplementary Figure 2. Regional division of China for domestic wastewater discharge patterns.** Based on the Technical Manual for Pollution Discharge Coefficients of Domestic Sources from China’s Second National Pollution Census, the country is divided into six regions. Each region is assumed to exhibit relatively homogeneous characteristics in terms of geography, climate, economic development, and water usage behaviors. Accordingly, a unified per capita hourly domestic wastewater discharge coefficient is applied within each region for modeling purposes (Supplementary Table 1).

**Supplementary Figure 3. Conceptual example for comparing TIDE and load rate.** Three conceptual regions (Region 1–3) are designed with identical total treatment capacity (2 units) but differing temporal and spatial patterns of influent. Region 1 experiences severe overloading during the day and no inflow at night. Region 2 maintains steady inflow, equally distributed across time. Region 3 includes two treatment plants with equal capacity: one is overloaded at all times, while the other remains idle. In all three cases, the traditional load rate metric equals 1, as total daily influent matches total design capacity. In contrast, the TIDE metric captures time–space mismatch and yields values of 0, 1, and 0 for Regions 1–3 respectively, demonstrating its superior ability to reflect true alignment between wastewater generation and treatment.

**Supplementary Figure 4. Assessment of decarbonisation potential in China. a** Intercity population mobility intensity across Chinese cities during 2021–2023, defined as the difference between the inflow index and outflow index on Lunar New Year’s Eve. **b** Daily average TIDE values across Chinese cities from 2021 to 2023. Calculation methods are detailed in the Methods. **c** Estimated improvements in daily average TIDE following the application of “gradient shutdown” and “flow equalisation” strategies across cities in China between 2021 and 2023. ΔTIDE was defined as the theoretical increase in TIDE achieved after applying the proposed strategies.

**Supplementary Figure 5. Population mobility in China on Spring Festival (2021–2023). a** City-level inflow index on Spring Festival from 2021 to 2023. **b** City-level outflow index on Spring Festival from 2021 to 2023.

**Supplementary Figure 6. Spatial distribution of alignment overestimation in China (2021–2023).** City-level overestimation of influent-capacity alignment is calculated as the daily difference between load rate and TIDE, divided by TIDE.

**Supplementary Figure 7. Treatment-inflow alignment and the population–TIDE relationship using the full dataset including rainfall days.** **a** Alignment between wastewater generation and treatment capacity using conventional load rate versus TIDE based on the full dataset. The gray diagonal line highlights the overestimation of alignment by the load rate metric. The gray shaded area denotes the 2023 Spring Festival travel period (2023.01.07–2023.02.15). **b** Positive correlation between daily population and TIDE in Shenzhen using the full dataset.

**Supplementary Figure 8. Linear relationships between population heat value and daily influent volume under full-data and rain-free conditions.** **a** Linear fit between population heat value and daily influent volume using the full dataset. **b** Linear fit between population heat value and daily influent volume after excluding rainfall days.

Supplementary Text S1 (Study areas):

This study is demonstrated in Shenzhen, a major megacity in southern China, and subsequently extended to a nationwide set of cities. The citywide analysis covered 35 of the 45 WWTPs officially operational within Shenzhen’s administrative boundaries by the end of 2023, which provided carbon emissions data (Supplementary Fig. 9). Detailed evaluation of targeted operational strategies—namely gradient shutdown and flow equalization—was conducted using operational data from a representative WWTP: the Longhua Phase 1 WWTP.

Shenzhen is located in the Guangdong–Hong Kong–Macao Greater Bay Area and borders Hong Kong to the south. As one of China’s four Tier-1 cities and its first Special Economic Zone, Shenzhen has experienced rapid urbanization and industrialization over the past four decades^15^. By the end of 2023, the city covered an area of approximately 2,000 km² and had a permanent population exceeding 17.7 million^16^. Shenzhen is characterized by high population density, frequent cross-district and intercity commuting, dense built-up areas, and high-water treatment demand. These features make it an ideal testbed for evaluating the dynamic behavior of wastewater systems under large-scale population mobility. To evaluate the generalizability of the proposed approach, we extended the analysis to the national scale by applying the TIDE indicator to 375 cities and regions across mainland China (as recorded in the 2023 China Urban Construction Statistical Yearbook).

**Supplementary Figure 9. Basin divisions and distribution of WWTPs in Shenzhen.** Shenzhen is divided into nine basin units based on its hydrological system and geographical features. Each basin serves as the basic unit for centralized wastewater management, overseen by sub-centers under the Shenzhen Water Authority. Red circles denote the 45 WWTPs that were constructed and operational by the end of 2023, with circle size proportional to their designed treatment capacity. The dark red circle highlights the Longhua Phase 1 WWTP, which serves as a case study for optimisation strategies and decarbonisation potential in the main text.

Supplementary Text S2 (Cross-city population decomposition):

To isolate how distinct cross-city commuting behaviours shape wastewater-sector GHG emissions, we developed an interpretable decomposition framework based on the Baidu population heat value, which proxies the intensity of people present within the city^17^. The framework uses only the hourly distribution of population heat value and does not require individual-level trajectory information, enabling the observed citywide population heat value time series to be decomposed into mutually exclusive components associated with different cross-city mobility behaviours (Supplementary Fig. 10). Because nighttime heat value patterns are less behaviourally interpretable, we restrict the analysis to the daytime window from 07:00 to 23:00, which fully covers both the morning and evening commuting peaks.

The decomposition is conducted on a weekly basis. For an arbitrary calendar week, let P_d_(t) denote the citywide population heat value at hour t∈[7,23] on day 𝑑 of that week (d=1,…,7). We decompose P_d_(t) into four mutually exclusive components associated with distinct cross-city behaviours: (i) a stable resident component S, representing individuals continuously present within the city throughout the study week with no in–out movement; (ii) a weekly outbound commuter component W_d_^out^(t), representing individuals who arrived before day d and leave the city on day d; (iii) a weekly inbound commuter component W_d_^in^(t), representing individuals who enter the city on day d and remain overnight; and (iv) a daily commuter component D_d_(t), representing individuals who both enter and leave the city within day d. Components (ii) and (iii) together constitute the weekly commuter population, denoted W_d_(t).

These components satisfy an hourly mass-balance (conservation) relationship:

$$P_{d}(t)=S+W_{d}^{out}(t)+W_{d}^{in}(t)+D_{d}(t)=S+W_{d}(t)+D_{d}(t)$$

The stable resident component S is assumed to be constant within the same calendar week, showing no variation across days or hours, and is defined as the minimum population heat value observed during that week. Analyses based on real population heat value data for Shenzhen indicate that this weekly minimum typically occurs at 07:00 on Sundays. Accordingly, S is computed as:

$$S=\min_{d,t\in\left[7,23\right]} P_{d}(t)$$

The weekly outbound commuter component $W_{d}^{\text{out }}\left( t \right)$ is already present in the city at 07:00 on day d and fully departs before 23:00 on the same day. Assuming an equal departure probability throughout 07:00–23:00, its size decreases linearly over time. We therefore construct a linear function $\tilde{P}_{d}\left( t \right)$ by connecting the total heat value at 07:00, P_d_ (7), and the stable component at 23:00, S, such that $\tilde{P}_{d}\left( 7 \right)=P_{d}\left( 7 \right)$ and $\tilde{P}_{d}\left( 23 \right)=S$. The hourly size of the weekly outbound component is then given by:

$$W_{d}^{out}(t)=\tilde{P}_{d}(t)-S$$

For each day d of the study week, we define the daily peak hour $t_{d}^{*}$ as the hour at which the population heat value reaches its maximum over the daytime window (07:00–23:00), i.e., $t_{d}^{*}=\arg\max_{t\in\left[ 7,23 \right]}P_{d}\left( t \right)$. The weekly inbound commuter component W_d_^in^(t) starts from zero at 07:00 on day d and increases as individuals enter the city. Because this group does not depart on the same day, we assume that its size remains constant after the daily peak hour t_d*. The magnitude of the inbound component at and after the peak is determined by closure at 23:00, using the difference between the total population heat value at 23:00 and the stable resident component S:

$$W_{d}^{in}(t)=W_{d}^{in}(23)=P_{d}(23)-S, t\in[t_{d}^{*},23]$$

After the daily peak $t_{d}^{*}$, the stable component and both weekly commuter components are fully determined; the daily commuter component is therefore obtained as the residual:

$$D_{d}(t)=P_{d}(t)-S-W_{d}^{out}(t)-W_{d}^{in}(t)$$

Before the peak $t_{d}^{*}$, we assume that daily commuters and weekly inbound commuters both start from zero at 07:00 and increase proportionally over time. The proportionality factor is defined using their sizes at the peak hour:

$$r_{d}=\frac{D_{d}(t_{d}^{*})}{W_{d}^{in}(t_{d}^{*})}$$

We define the newly added population intensity $A_{d}\left( t \right)$ as the portion jointly contributed by daily commuters and weekly inbound commuters after removing the stable component and the weekly outbound component:

$$A_{d}(t)=P_{d}(t)-S-W_{d}^{out}(t)$$

Accordingly, $A_{d}\left( t \right)$ is partitioned as:

$$W_{d}^{in}(t)=\frac{A_{d}(t)}{1+r_{d}}, D_{d}(t)=A_{d}(t)-W_{d}^{in}(t)$$

Through the above procedure, the four population components over 07:00–23:00 can be uniquely determined at an hourly resolution for each day of the study week while satisfying the conservation relationship. For the analysis in Fig. 3, the weekly outbound and weekly inbound components are aggregated and reported as a single weekly commuter component.

**Supplementary Figure 10. Decomposition of the urban population.** **a** The total urban population is decomposed into four mutually exclusive components: stable population, weekly inbound population, weekly outbound population, and daily commuting population; the weekly inbound and outbound components are collectively referred to as the weekly commuting population. **b** Within the time window from 7:00 to 23:00, which fully covers the morning and evening peak hours, the four population categories exhibit distinct temporal dynamics. The stable population remains constant throughout the day, the weekly outflow population decreases linearly, while both the weekly inflow population and the daily commuting population increase proportionally from zero to the daily peak. After the peak, the weekly inflow population remains constant, whereas the daily commuting population gradually decreases to zero.

Supplementary Text S3 (Mobility-enhanced Influent Forecasting):

Low-carbon operational optimisation requires accurate influent forecasting, particularly at multi-hour horizons that provide sufficient lead time for scheduling decisions. Previous studies have primarily relied on time-series approaches that extrapolate periodic patterns from historical influent records to anticipate short-term trends^18,19^. However, complex human mobility behaviours—such as commuting, tourism, and business travel—introduce substantial uncertainty into influent dynamics^20^. Without mobility information, further improvements in existing influent forecasting models remain constrained. Here we incorporate population heatmap data as a proxy for the spatial distribution of people and use it as an additional input feature for influent prediction. We evaluate its contribution to forecasting performance across 27 wastewater service area in Shenzhen, which serve as the analysis units because wastewater can be redistributed among WWTPs within the same service area.

Both the baseline and mobility-enhanced models were built on the long short-term memory (LSTM) architecture, a widely used approach for influent forecasting^21,22^, and were implemented in Python using PyTorch library. Using influent flow observations from 1 January to 31 October 2023 as the primary dataset, we split the samples into training, validation, and test sets at a 13:4:3 ratio. This split was designed to ensure that each subset covered complete natural workweeks as well as major public holidays (i.e., the Spring Festival, Labour Day, and the National Day holiday), thereby reflect the effects of population mobility on the wastewater system. The two model settings employed identical hyperparameter ranges (Supplementary Table 5) and differed only in whether mobility-derived features were included in the input. The hyperparameter ranges were determined through multiple univariate experiments. Model training used an early-stopping scheme, with training terminated when the validation loss did not decrease for 40 consecutive epochs, and a maximum of 500 epochs. We used Adam as the learning rate optimizer and ReLU as the activation function. Forecasting was conducted under a sliding-window setup, and performance was evaluated on the test set for 1-, 3-, and 7-step-ahead predictions.

**Supplementary Table 5. Selection range for hyperparameters**

| Hyperparameters | Selection ranges |
| --- | --- |
| Loss function | MSE, Weighted MSE (α = 5.0, 10.0, 20.0) |
| Batch size | 16, 32, 64, 128, 256 |
| Number of hidden layers | 1, 2, 3 |
| Number of neurons in hidden layer | 16, 32, 64, 128, 256, 512 |

The results show that incorporating population information consistently improves influent forecasting performance across WWTP service areas and forecasting horizons (Supplementary Fig. 11). For 1-, 3-, and 7-step-ahead predictions, 81.5%, 85.2%, and 74.1% of service areas exhibited higher test-set R² values when mobility features were included, with average improvements of 1.3%, 10.9%, and 17.9%, respectively (Supplementary Table 6). These findings indicate that the performance gains enabled by population mobility information are robust and broadly applicable. As the forecasting horizon extends, conventional time-series-based models show decreasing predictive performance, whereas the benefit of mobility features becomes more pronounced. This divergence underscores the value of population-informed forecasting for longer lead times and provides a reliable foundation for implementing the proposed low-carbon operational strategies in WWTPs.

**Supplementary Table 6. Comparison of influent forecasting performance with and without mobility features**

|  | 1 hour | | | 3 hour | | | 7 hour | | |
| --- | --- | --- | --- | --- | --- | --- | --- | --- | --- |
| service area | R^2^_0 | R^2^­_1 | ΔR^2^(%) | R^2^_0 | R^2^­_1 | ΔR^2^(%) | R^2^_0 | R^2^­_1 | ΔR^2^(%) |
| Kui Yong | 0.75 | 0.78 | 4.5% | 0.37 | 0.51 | 38.9% | 0.28 | 0.44 | 56.9% |
| Xi Li | 0.56 | 0.58 | 3.5% | 0.51 | 0.62 | 22.3% | 0.39 | 0.49 | 23.9% |
| Shui Tou | 0.58 | 0.60 | 3.2% | 0.45 | 0.55 | 20.6% | 0.41 | 0.50 | 21.5% |
| Bu Ji | 0.88 | 0.89 | 1.9% | 0.62 | 0.71 | 14.5% | 0.48 | 0.57 | 17.8% |
| Guang Ming | 0.79 | 0.81 | 2.1% | 0.10 | 0.18 | 75.4% | 0.51 | 0.59 | 15.5% |
| Long Hua | 0.85 | 0.87 | 1.9% | 0.57 | 0.63 | 11.5% | 0.52 | 0.59 | 14.8% |
| Pu Dixia | 0.84 | 0.86 | 1.8% | 0.54 | 0.59 | 9.1% | 0.60 | 0.66 | 10.8% |
| Bin He | 0.82 | 0.84 | 1.6% | 0.73 | 0.77 | 5.7% | 0.65 | 0.71 | 9.9% |
| E Gongling | 0.89 | 0.90 | 1.1% | 0.65 | 0.69 | 6.4% | 0.40 | 0.46 | 15.3% |
| Yan Tian | 0.76 | 0.77 | 1.2% | 0.62 | 0.67 | 6.6% | 0.05 | 0.11 | 116.3% |
| Fu Tian | 0.89 | 0.90 | 1.0% | 0.38 | 0.42 | 8.2% | 0.61 | 0.67 | 9.8% |
| Long Tian | 0.89 | 0.90 | 0.8% | 0.40 | 0.43 | 6.9% | 0.70 | 0.76 | 7.8% |
| Fu Yong | 0.88 | 0.89 | 0.6% | 0.76 | 0.78 | 3.7% | 0.55 | 0.60 | 8.3% |
| Nan Shan | 0.83 | 0.84 | 0.6% | 0.69 | 0.72 | 3.9% | 0.75 | 0.79 | 5.8% |
| Heng Ling | 0.84 | 0.84 | 0.5% | 0.67 | 0.70 | 3.3% | 0.74 | 0.77 | 4.7% |
| Sha Tian | 0.82 | 0.82 | 0.5% | 0.62 | 0.64 | 2.5% | 0.73 | 0.76 | 4.4% |
| Ban Xuegang | 0.89 | 0.89 | 0.4% | 0.33 | 0.35 | 4.4% | 0.35 | 0.38 | 8.3% |
| Guan Lan | 0.91 | 0.91 | 0.4% | 0.82 | 0.83 | 1.6% | 0.48 | 0.49 | 3.2% |
| Sha Jing | 0.94 | 0.94 | 0.2% | 0.79 | 0.80 | 1.2% | 0.57 | 0.58 | 1.9% |
| Luo Fang | 0.92 | 0.92 | 0.1% | 0.76 | 0.77 | 1.1% | 0.80 | 0.80 | 1.1% |
| Gu Shu | 0.74 | 0.74 | 0.1% | 0.82 | 0.83 | 0.8% | 0.37 | 0.37 | -0.2% |
| Shang Yang | 0.83 | 0.83 | 0.1% | 0.76 | 0.77 | 0.7% | 0.74 | 0.74 | -0.3% |
| Ping Hu | 0.83 | 0.83 | -0.4% | 0.81 | 0.81 | 0.6% | 0.70 | 0.68 | -1.9% |
| Gong Ming | 0.64 | 0.64 | -0.6% | 0.76 | 0.75 | 0.0% | 0.11 | 0.09 | -12.5% |
| Yan Chuan | 0.60 | 0.59 | -0.7% | 0.67 | 0.67 | -0.6% | 0.72 | 0.69 | -4.2% |
| She Kou | 0.81 | 0.80 | -1.1% | 0.46 | 0.46 | -1.7% | 0.43 | 0.40 | -7.3% |
| Heng Gang | 0.67 | 0.64 | -4.2% | 0.55 | 0.53 | -3.1% | 0.44 | 0.32 | -26.4% |

R²_0 and R²_1 denote the test-set R² values of the models without and with population information, respectively.

**Supplementary Figure 11. Performance gains from population information across multiple forecasting horizons.** Performance gains are quantified as the relative increase in R², calculated as the difference between R² values with and without population information, normalized by the baseline R² without population information. **a–c**, Relative R² improvements for 1 h, 3 h, and 7 h ahead influent forecasts, respectively.

Supplementary Text S4 (Optimisation Threshold Selection):

To reduce the carbon intensity of wastewater treatment, we designed a flow equalization strategy that redistributes influent across time—storing wastewater during peak hours and treating it during off-peak periods. In doing so, it also mitigates overload conditions and reduces the risk of overflow, thereby enhancing operational stability.

However, it is not necessary to implement this strategy on every single day. On some days, hourly influent volumes remain well within the design capacity, and the overall treatment-generation alignment is already high. In such cases, flow equalization would yield limited additional benefit and may unnecessarily increase operational complexity. Therefore, an optimisation threshold is required to determine when the strategy should be applied. To determine an appropriate threshold, we evaluated seven TIDE improvement benchmarks—0%, 5%, 10%, 20%, 30%, 40%, and 50%—and examined their corresponding outcomes in terms of average TIDE improvement, carbon reduction, and the proportion of optimisation-eligible days among all rain-free days. These results are summarized in Supplementary Table 7.

**Supplementary Table 7. Evaluation of threshold settings for flow equalization implementation**

| Optimisation threshold | TIDE improvement | Carbon  reduction | Optimisation  days |
| --- | --- | --- | --- |
| 0% | 15.6% | 6.5% | 100.0% |
| 5% | 26.7% | 12.0% | 55.6% |
| 10% | 34.8% | 14.6% | 39.4% |
| **20%** | **46.6%** | **17.5%** | **24.9%** |
| 30% | 75.6% | 21.6% | 10.8% |
| 40% | 168.0% | 29.3% | 3.3% |
| 50% | 185.7% | 30.8% | 2.9% |

As illustrated in Supplementary Fig. 12, both TIDE and carbon reduction benefits increase with higher optimisation thresholds. However, the relationship between TIDE improvement and carbon reduction follows a clear logarithmic trend, indicating diminishing marginal returns. In other words, the efficiency of carbon mitigation gains plateaus as the threshold rises. Simultaneously, the number of days eligible for optimisation decreases sharply with stricter threshold settings.

Based on this trade-off analysis, we selected a 20% TIDE improvement threshold as the optimal balance. It achieves a notable average carbon reduction of 17.5%, while still allowing optimisation on 24.9% of all rain-free days. This setting captures most of the mitigation potential while maintaining practical applicability.

**Supplementary Figure 12. Performance under different TIDE optimisation thresholds.** Seven TIDE improvement thresholds ranging from 0% to 50% were evaluated and color-coded accordingly. A clear logarithmic relationship (r^2^ = 0.99928) was observed between TIDE improvement and carbon reduction potential across different thresholds.

Supplementary Text S5 (Sensitivity analysis of TIDE):

TIDE is calculated from hourly influent flow, hourly design capacity and plant-scale weighting. Therefore, its value may be affected by uncertainty in influent-flow observations, design-capacity settings, and the weighting scheme used when multiple WWTPs are aggregated. To evaluate the sensitivity of TIDE to these key inputs, we conducted a one-at-a-time perturbation analysis using the Shenzhen plant-level dataset.

Hourly influent flow *q* and design capacity *d* were perturbed by ±10%, while the other variables were kept unchanged. For the scale-weighting term *ω*, two alternative weighting schemes were tested: equal weighting across all WWTPs and square-root capacity weighting. The latter weakens the dominance of large WWTPs while still retaining plant-scale information. Sensitivity was quantified as the mean absolute daily change in TIDE relative to the baseline:

$$Mean Absolute Change=\frac{1}{n}\sum_{d=1}^{n} |TIDE_{scenario,d}-TIDE_{baseline,d}|$$

where *n* is the number of analysed days, and *d* denotes the *d*-th analysed day.

The results show that TIDE was mainly sensitive to the flow–capacity terms *q* and *d* (Supplementary Table 8). Perturbing *q* and *d* by ±10% resulted in mean absolute changes of 0.0316–0.0356. In contrast, changing the weighting scheme caused smaller deviations, with mean absolute changes of 0.0111 under equal weighting and 0.0059 under square-root capacity weighting. These results indicate that TIDE is primarily driven by the deviation between hourly influent flow and design capacity, whereas the city-level TIDE estimate is relatively robust to the specific scale-weighting scheme.

**Supplementary Table 8. Sensitivity analysis of TIDE**

| Perturbed term | Scenario | Mean TIDE | Mean absolute change in TIDE |
| --- | --- | --- | --- |
| -- | Baseline | 0.7365 | -- |
| *q* | *q* × 0.9 | 0.7173 | 0.0356 |
| *q* | *q* × 1.1 | 0.7298 | 0.0316 |
| *d* | *d* × 0.9 | 0.7279 | 0.0351 |
| *d* | *d* × 1.1 | 0.7204 | 0.0320 |
| *ω* | Equal weight | 0.7329 | 0.0111 |
| *ω* | Square-root capacity weight | 0.7359 | 0.0059 |

Supplementary Text S6 (Strategy Feasibility):

We used Guanlan WWTP (Phase 2) in the Guanlan River Basin as an example to assess the feasibility of the proposed strategy. The plant has a design capacity of 240,000 m^3^/day, employs a modified A^2^/O biochemical process, and possesses complete hourly influent monitoring data for 2023, sufficient to support feasibility analysis.

Supplementary Fig. 13 illustrates the conceptual calculation of required storage volume and maximum stored water duration. The core idea of the flow equalisation strategy is: when the actual influent flow in an hour exceeds the target balanced treatment flow, the excess is temporarily stored; when below the target, stored water is released.

**Supplementary Figure 13. Conceptual calculation of storage volume and duration for flow equalisation. a** Hourly influent flow is compared with the target equalised flow $Q_{target}$. When influent flow exceeds $Q_{target}$, the excess flow is temporarily stored; when influent flow is below $Q_{target}$, stored water is released to supplement treatment. **b** The required storage volume $V_{d}$is determined by the maximum fluctuation range of stored volume within day *d*, while the maximum storage duration $T_{d}$ is approximated by the longest continuous storage–release cycle.

For day *d*, the target hourly treatment flow is defined as:

$$Q_{target,d}= \frac{1}{24}\sum_{h=1}^{24} Q_{d,h}$$

where $Q_{d,h}$ is the actual influent flow in hour *h* of day *d*. The cumulative stored water volume is:

$$C_{d,t}=\sum_{h=1}^{t} (Q_{d,h}-Q_{t\arg et,d})$$

An increasing $C_{d,t}$ indicates storage of excess inflow, while a decreasing $C_{d,t}$ indicates release. The minimum storage tank volume for day *d* is determined by the maximum range of $C_{d,t}$:

$$V_{d}=\max_{t} (C_{d,t})-\min_{t} (C_{d,t})$$

The yearly minimum storage volume is the maximum of daily $V_{d}$, i.e., $V_{d,max}$.

Maximum hydraulic retention time is approximated by the longest continuous storage period $T_{d}=t_{e}-t_{s}$, where $t_{s}$is the start of storage and $t_{e}$is the end of storage. Measured data from Guanlan Phase 2 indicate that there is typically one storage cycle per day.

Based on measured hourly data for 2023 from Guanlan WWTP (Phase 2), we calculated daily $V_{d}$and $T_{d}$, as well as annual maxima $V_{d,\max}$and $T_{d,\max}$, shown in Supplementary Table 9. The maximum storage volume $V_{d,\max}$was 3.68×10^4^ m^3^, corresponding to 14.7% of the plant’s design capacity. The maximum storage duration $T_{d,\max}$was 20 h, with an average duration of 15.45 h. These results indicate that, under extreme conditions, the storage could last for a relatively long period. Therefore, practical implementation should include appropriate mixing and odour control, with low-intensity aeration if necessary, to prevent water deterioration. It should be noted that $T_{d}$represents the theoretical maximum storage duration, i.e., the time from when the earliest portion of water is stored until it is treated, and the average storage time is generally shorter than this value.

**Supplementary Table 9. Storage requirement and duration at Guanlan WWTP Phase 2.**

| Metric | Date | Value | Unit | Notes |
| --- | --- | --- | --- | --- |
| $V_{d,\max}$ | 2023-08-10 | 3.68 | 10^4^m³ | Maximum daily storage volume |
| $V_{d,\mathrm{mean}}$ | 2023 | 1.61 | 10^4^m³ | Average daily storage volume |
| $T_{d,\max}$ | 2023-07-16 | 20.00 | h | Maximum storage duration |
| $T_{d,\max}$ | 2023 | 15.45 | h | Average daily storage duration |

In engineering practice, some WWTPs or their upstream drainage systems have already been equipped with storage facilities. For example, the Guanlan storage tank, located upstream of Guanlan WWTP Phase 2, was completed in 2013. It occupies an area of 3.2×10^4^ m^2^, has a storage volume of 2.19×10^5^ m^3^, and has a pumping capacity of 4.0×10^5^ m^3^/d. Its original functions included initial-rainwater storage and emergency storage during accidental shutdown or reduced operation of the WWTP. In terms of storage capacity and functional positioning, this facility has the potential to support the proposed flow equalisation strategy.

The Standard for Design of Outdoor Wastewater Engineering (GB 50014—2021) has also incorporated storage facilities as formal engineering units in outdoor drainage-system design. Its explanatory notes further indicate that stormwater storage tanks can be installed at suitable locations for runoff peak control and for reducing pressure on downstream drainage pipelines. This suggests that using storage facilities to balance short-term flow fluctuations and relieve pressure on downstream treatment systems is an established concept in drainage engineering. Therefore, for WWTPs that already have storage facilities, the existing storage capacity should first be evaluated for potential use in flow equalisation. For WWTPs without sufficient storage capacity, the scale and cost of newly built or expanded storage facilities should be further assessed.

Here, we used Guanlan WWTP Phase 2 as an example to estimate the construction cost required if a new storage tank were constructed according to the flow equalisation demand. Based on the above calculation, the storage volume required to meet the maximum equalisation demand was 3.68×10^4^ m^3^. We collected publicly available storage-tank construction projects in China and derived the unit construction cost from the reported storage volume and investment of each project, as shown in Supplementary Table 10. The unit costs of the reference projects ranged from 1331 to 5852 CNY/m³, with a mean value of 2957 CNY/m³. Accordingly, the preliminary construction cost of the required storage tank at Guanlan WWTP Phase 2 was estimated as:

$$Cost = V_{d,\max}\times Unit\text{ }cost=3.68\times10^{4}\times2957\approx1.09\times10^{8}\text{CNY}$$

This estimate should be interpreted as a preliminary order-of-magnitude construction-cost estimate based on comparable public engineering cases. In practical implementation, site-specific cost accounting should further consider land occupation, tank burial depth, mixing, odour control, automation, management, and other engineering conditions.

**Supplementary Table 10. Reference storage-tank projects used for construction-cost estimation.**

| Project | Storage volume (10^4^m³) | Cost  (CNY) | Unit cost (CNY/m³) |
| --- | --- | --- | --- |
| Shangxiacun storage tank^23^ | 29 | 386 million | 1331 |
| Xixia District central drainage-system storage tank^24^ | 3 | 66.2 million | 2208 |
| Wencui North Street storage tank^25^ | 5 | 132.8 million | 2657 |
| Nanjing Road storage tank^26^ | 4 | 234.1 million | 5852 |
| Mean | -- | -- | 2957 |

SI References

1.	Yang, H. Investigation on intraday variation of illicit drugs in municipal wastewater and drug abuse during New Year holiday. Dalian Maritime University, 2022.

2.	Zheng, H. Analysis on the variation law of domestic sewage discharge in Chaoyang City. *Technology of Soil and Water Conservation*, 4-5 (2024).

3.	Niu, Y. et al. Wastewater quantity and quality fluctuation characteristics of drainage system in Tuandao, Qingdao. *Environmental Engineering*. **39**, 18-24 (2021).

4.	Jiang, S. et al. Study on pollutant generation coefficient of urban domestic sewage. *Water & Wastewater Engineering*. **53**, 86-89 (2017).

5.	Zhao, P. & Xu, J. Research on the emission laws of urban domestic sewage in northwest area——Taking Lanzhou City as an example. *Journal of Northwest Normal University (Natural Science)*. **57**, 18-23 (2021).

6.	Xu, J. Study on pollutant emission law and, Coefficient of urban domestic source water in Gansu Province. Lanzhou University, 2019.

7.	Sun, Y. et al. Emission Regularity of Daily Domestic Sewage Pollutant Production of Urban Residents Per Capita. *China Water & Wastewater*. **36**, 1-6 (2020).

8.	Zheng, Y., Zheng, L., Wu, C. & Zhang, C. Characteristics of domestic wastewater quality and quantity discharged of Zhenjiang city. *Journal of Jiangsu University of Science and Technology (Natural Science Edition)*. **26**, 91-94 (2012).

9.	Wu, B. et al. Discharge law of domestic wastewater and pollutants discharge of urban residents in south China. *Environmental Engineering*. **40**, 196-201 (2022).

10.	Li, H. et al. Measurement method and application of domestic sewage pollutant concentration in high-density built-up area in Southern. *Water & Wastewater Engineering*. **59**, 53-59 (2023).

11.	Wu, X., Zhang, Y., He, J., Zhou, H. & Zheng, W. Characteristics of Urban Residents Domestic Sewage in Dianchi Lake Watershed. *China Water & Wastewater*. **37**, 89-95 (2021).

12.	Yang, X. The Discharge Characteristics and Influence Factors of Domestic Wastewater in Residential Communities in the Main Urban Area of Chongqing. Chongqing University, 2020.

13.	Handbook of Calculation Methods and Coefficients for Emission Source Statistics and Investigation (Ministry of Ecology and Environment of the People's Republic of China,2021); https://www.mee.gov.cn/xxgk2018/xxgk/xxgk01/202106/t20210618_839512.html

14.	Parravicini, V., Svardal, K. & Krampe, J. Greenhouse Gas Emissions from Wastewater Treatment Plants. *Energy Procedia*. **97**, 246-253 (2016).

15.	Cheng, J., Chen, M. & Tang, S. Shenzhen-A typical benchmark of Chinese rapid urbanization miracle. *Cities*. **140**, 14 (2023).

16.	*Statistical Bulletin of Shenzhen's National Economic and Social Development in 2023.*

(Statistics Bureau of Shenzhen Municipality, 2024);

https://tjj.sz.gov.cn/gkmlpt/content/11/11264/post_11264245.html#4222

17.	Wang, J. et al. Transforming Noise Control Stations into Soundscape Resource Monitoring Networks: An Adaptive Location Recommendation. *Environ. Sci. Technol.* **59**, 21527-21539 (2025).

18.	Zhou, P., Li, Z., Zhang, Y., Snowling, S. & Barclay, J. Online machine learning for stream wastewater influent flow rate prediction under unprecedented emergencies. *Front. Env. Sci. Eng.* **17**, 11 (2023).

19.	Cheng, T., Harrou, F., Kadri, F., Sun, Y. & Leiknes, T. Forecasting of Wastewater Treatment Plant Key Features Using Deep Learning-Based Models: A Case Study. *IEEE Access*. **8**, 184475-184485 (2020).

20.	Zhong, L., Dong, L., Wang, Q. R., Song, C. & Gao, J. Universal expansion of human mobility across urban scales. *Nature Cities*. **2**, 12 (2025).

21.	Duarte, M. S. et al. A Review of Computational Modeling in Wastewater Treatment Processes. *Acs Es&T Water*. **4**, 784-804 (2023).

22.	Hochreiter, S. & Schmidhuber, J. Long short-term memory. *Neural Comput.* **9**, 1735-1780 (1997).

23.	*China Water and Electricity 11th Bureau won the bid for the Shangxia Village Regulation Pond Project of the Comprehensive Water Environment Improvement Project in Maozhou River Basin, Shenzhen.* (Power Construction Corporation of China, 2018); https://mhuanbao.in-en.com/html/huanbao-2283898.shtml

24.	*Announcement on the Proposed Construction Project of Regulation Ponds for the Central Drainage System in Xixia District, Yinchuan.* (Yinchuan Municipal People's Government, 2023); https://www.yinchuan.gov.cn/xxgk/bmxxgkml/ycsszglj_38951/xxgkml_38954/szjcssjsjwh/202310/t20231031_4333600.html

25.	*Public Participation Announcement for Social Stability Risk Assessment of the Construction Project of Regulation and Storage Ponds on North Wencui Street, Central Xixia District, Yinchuan.* (Yinchuan Municipal Bureau of Housing and Urban-Rural Development, 2025); https://zjj.yinchuan.gov.cn/zwgk/fdzdgknr/gsgg/ghgs/202502/t20250210_4816033.html

26.	*Phase III Project of Rainwater Regulation and Storage Pond (Nanjing Road Regulation and Storage Pond).* (Tianjin Public Resources Trading Service Platform, 2026); https://ggzyfw.beijing.gov.cn/xtggtjzbjh/20260226/5436686.html?
